# The Performance of Artificial Intelligence in Classifying Molecular Markers in Adult-Type Gliomas Using Histopathological Images: Systematic Review

**DOI:** 10.2196/78377

**Published:** 2026-03-13

**Authors:** Obada Almaabreh, Rukaya Al-Dafi, Aliya Tabassum, Ahmad Othman, Alaa Abd-alrazaq

**Affiliations:** 1 Faculty of Medicine, Yarmouk University, Irbid, Jordan; 2 Department of Computer Science and Engineering, College of Engineering, Qatar University, Doha, Qatar; 3 AI Center for Precision Health, Weill Cornell Medical College in Qatar, 103 Ezdan Oasis, Alwakrah, Doha, Qatar, 974 17849573; 4 Department of Biomedical Sciences, College of Health Sciences, Qatar University, Doha, Qatar

**Keywords:** glioma, brain tumors, molecular markers, artificial intelligence, histopathology, isocitrate dehydrogenase, IDH mutation, 1p/19q codeletion, systematic review

## Abstract

**Background:**

Adult-type gliomas are among the most prevalent and lethal primary central nervous system tumors, where prompt and accurate diagnosis is essential for maximizing survival prospects. Molecular classification, particularly the detection of isocitrate dehydrogenase (IDH) mutations and 1p/19q codeletions, has become crucial for accurate diagnosis and prognosis. Artificial intelligence (AI) has emerged as a promising adjunct in enhancing diagnostic accuracy using histopathological images. Existing reviews mostly focused on radiology rather than histopathology, and no comprehensive systematic review has specifically evaluated AI performance exclusively from histopathological images for detecting these two molecular markers.

**Objective:**

This study aims to systematically evaluate the performance of AI models in detecting and classifying IDH mutation status and 1p/19q gene codeletion in adult-type gliomas using histopathological images.

**Methods:**

A systematic review was conducted in accordance with PRISMA-DTA (Preferred Reporting Items for Systematic Reviews and Meta-Analyses–Extension for Diagnostic Test Accuracy) guidelines. Seven databases (MEDLINE, PsycINFO, Embase, IEEE Xplore, ACM Digital Library, Scopus, and Google Scholar) were searched for studies published between 2015 and 2025. Eligible studies used AI models on histopathological images for molecular classification of adult-type gliomas and reported performance metrics. Study selection, data extraction, and risk of bias assessment using a modified QUADAS-2 (Quality Assessment of Diagnostic Accuracy Studies 2) tool were conducted independently by two reviewers. Extracted data were synthesized narratively.

**Results:**

A total of 2453 reports were identified, with 22 studies meeting the inclusion criteria. The pooled average accuracy, sensitivity, specificity, and area under the curve (AUC) across studies were 85.46%, 84.55%, 86.03%, and 86.53%, respectively. Hybrid models demonstrated the highest diagnostic performance (accuracy 92.80% and sensitivity 89.62%). In general, AI models that used multimodal data outperformed those that used unimodal data in terms of sensitivity (90.15% vs 84.31%) and AUC (88.93% vs 86.29%). Furthermore, models had a better overall performance in identifying IDH mutations than 1p/19q codeletions, with higher accuracy (86.13% vs 81.63%), specificity (86.61% vs 78.11%), and AUC (86.74% vs 85.15%). Unexpectedly, AI models designed for binary classification exhibited lower performance than those for multiclass classification in terms of both accuracy (91.98% vs 84.02%) and sensitivity (93.41% vs 80.18%). However, these differences should be interpreted as descriptive trends rather than statistically validated superiority, as formal between-group comparisons were not feasible.

**Conclusions:**

AI models show strong potential as complementary tools for the molecular classification of adult-type gliomas using histopathology images, particularly for IDH mutation detection. However, these findings are constrained by the limited number of studies, the focus on adult-type gliomas, lack of meta-analysis, and restriction to English-language publications. While AI offers valuable diagnostic support, it must be integrated with expert clinical judgment. Future research should prioritize larger, more diverse datasets and multimodal AI frameworks and extend to other brain tumor types for broader applicability.

## Introduction

### Background

Gliomas are the most frequent primary tumors affecting the central nervous system (CNS) in adults, with a variable prognosis depending on the specific subtype and histological grade [1]. The global prevalence of CNS neoplasms in 5 years is approximately 771,110 cases. Primary brain tumors account for 1.7% of all cancers, with a global incidence of 3.9 cases per 100,000 people each year. Among these, gliomas are the most common malignant type, accounting for 75% of all malignant CNS tumors, with an incidence of 6 cases per 100,000 people annually [2]. Gliomas are among the tumors that are difficult to treat, with a 5-year overall survival of less than 35% [3]. Patients with brain tumors present with variable clinical symptoms according to the part of the brain affected. However, they usually share general symptoms that are nonspecific to anatomic location (eg, seizures, headaches).

Gliomas are neuroectodermal tumors arising from glial cells or their precursors and encompass astrocytomas, oligodendrogliomas, and ependymomas [3]. Mutations in isocitrate dehydrogenase 1 and 2 (IDH1 and IDH2) are considered early events in gliomagenesis and occur more frequently in lower-grade gliomas [3]. Diffuse gliomas harboring IDH1/2 mutations are associated with significantly improved prognosis compared with IDH–wild-type diffuse gliomas [3].

Codeletion of chromosomal arms 1p and 19q results from an unbalanced centromeric translocation, t(1;19)(q10;p10) [3]. In combination with IDH mutation, 1p/19q codeletion is a defining molecular feature required for the diagnosis of oligodendroglioma, IDH mutant, and 1p/19q codeleted. This molecular alteration is associated with a favorable prognosis among diffuse gliomas and predicts enhanced responsiveness to alkylating chemotherapy [3].

Conventional classification of gliomas relied on histologic features as a gold standard. Initially, pathologists utilized microscopic analysis of histochemically stained (eg, hematoxylin and eosin) sections for the diagnosis of gliomas [4]. However, ancillary immunohistochemistry has been increasingly used over the past few decades to enhance diagnostic accuracy [4]. With either method, pathologists followed a diagnostic algorithm to rule out nonneoplastic lesions (eg, infarcts) or other types of malignancies (eg, metastatic) based on histologic features. Clinical information (eg, age) and radiologic findings (eg, location) are also valuable clues in the diagnosis [4]. Nonetheless, the histologic-based classification system has a few limitations, particularly interobserver variability, in addition to insufficient or nonrepresentative tissue sampling [5]. Although histologic-based classification can be accurate for prototypic tumors, classification of tumors encountered in clinical practice is often more challenging due to a degree of mixed features in the same sample [5].

In order to address these limitations, molecular-based diagnosis has emerged as a possible solution. Recent advancements in genetics have helped to identify specific gene alterations involved in the development of different types of gliomas. Detection of these gene alterations is the base of molecular-based diagnosis. This method offers improved accuracy and made it possible to distinguish between primary and secondary gliomas, which was not possible with the previous histologic-based method [6]. Therefore, the World Health Organization (WHO) integrated molecular features into its most recent classification of CNS tumors, published in 2021, based on the presence or absence of IDH1 and IDH2 mutations, in addition to chromosome 1p/19q codeletion [7]. It categorized adult-type gliomas into three categories: astrocytoma with IDH mutation, oligodendroglioma with IDH mutation and 1p/19q codeletion, and glioblastoma with IDH wild-type [7]. Furthermore, a combined approach using molecular and histologic features is used for the grading of IDH-mutant astrocytomas as CNS WHO grade 2, 3, or 4. The new WHO classification also led to a more accurate prognosis of gliomas, as the absence of IDH mutation is often a sign of more aggressive tumors [7].

Neuroimaging provides noninvasive insight into the molecular landscape of diffuse gliomas. Advanced magnetic resonance imaging (MRI) features can suggest IDH mutation status, as IDH-mutant tumors typically exhibit lower perfusion and less aggressive radiological behavior than IDH–wild-type gliomas [5-7]. Magnetic resonance spectroscopy allows in vivo detection of the IDH-specific oncometabolite D-2-hydroxyglutarate, enabling preoperative identification of IDH-mutant tumors [7]. In addition, oligodendrogliomas defined by combined IDH mutation and 1p/19q codeletion display characteristic imaging features, including cortical localization, calcifications, and relatively increased perfusion, reflecting distinct tumor biology [4,7]. These radiogenomic associations support the integration of imaging into molecular stratification of adult-type diffuse gliomas [4-6].

As gliomas pose diagnostic challenges due to their complex histological and molecular features, artificial intelligence (AI) has emerged as a promising tool to enhance diagnostic accuracy and efficiency. AI models were able to offer rapid and accurate results with accuracy exceeding 90%, which can further improve the access and speed of molecular diagnosis [8]. In general, AI implementation in health care can lead to improved data synthesis (eg, patient data, medical literature) [9]. Additionally, AI can process multimodal data (two or more different data sources such as histopathological images, radiological data, clinical variables, genomic markers, and other relevant data types) to provide more accurate diagnosis and predictions. Moreover, it offers means for augmenting human performance, as human limitations can prevent processing large quantities of information and making the optimal judgment at all times. Thus, AI can improve care consistency, increase precision, accelerate discoveries, and minimize disparities [9].

### Research Gaps and Aim

The use of AI in the diagnosis of brain tumors and gliomas has been widely studied, with many reviews summarizing the results of numerous studies. However, existing reviews exhibit several limitations. For instance, many reviews focused on AI implementation in other diagnostic methods of gliomas, such as radiology-based [10,11], surgery-based [12], and DNA-based diagnoses [13]. As for reviews focusing on AI implementation in the molecular diagnosis of gliomas, they fall into one of the following categories: not systematic [14], outdated [15], restricted by databases or search terms [11,16], lacking subtyping of gliomas [17], not focused on genetic markers (IDH mutations and 1p/19q codeletion) [11], or limited to a specific subfield of AI (eg, deep learning or machine learning) [16]. To address the limitations of the previous reviews, this study aims to systematically assess the performance of AI in detecting and classifying IDH mutation status and 1p/19q gene codeletion in adult-type gliomas using histopathological images.

## Methods

This review was conducted in accordance with the PRISMA-DTA (Preferred Reporting Items for Systematic Reviews and Meta-Analyses–Extension for Diagnostic Test Accuracy) [18] (see Checklist 1) and registered with the international Prospective Register of Systematic Reviews (PROSPERO) under the number CRD420250653668.

### Search Strategy

The literature search was performed on November 15, 2024, using the following digital databases: MEDLINE, PsycINFO, Embase, IEEE Xplore, ACM Digital Library, Scopus, and Google Scholar. A biweekly automated search was set for 4 months, concluding on March 15, 2025. Given the large number of results from Google Scholar, which are ranked by relevance, we reviewed only the first 100 entries (10 pages). Our query utilized a combination of proper keywords and Boolean operators, including but not limited to ((“Artificial Intelligence” OR “Machine Learning” OR “Deep Learning”) AND (“Histopatholog*” OR “Whole-Slide Imag*”) AND (“Glioma*” OR “Astrocytoma*” OR “Oligodendroglioma*” OR “Glioblastoma*”)). The search was restricted to studies published between 2015 and 2025. The search query for each database is shown in Multimedia Appendix 1. To identify additional studies, we screened the reference lists of included studies (ie, backward reference list checking) and checked studies that cited the included studies (ie, forward reference list checking).

### Study Selection

The selection process of relevant studies consisted of three steps. The first step involved removing duplicates using EndNote. In the second step, the studies were assigned to two authors, who screened the papers independently by title and abstract. The final step involved each author reading the consequent papers in full text independently. Discussions were held to resolve any differences or ambiguities in steps two and three.

### Study Eligibility Criteria

Studies fulfilling the following criteria were included: (1) original articles, theses, dissertations, and conference papers written in English, (2) studies published in 2015 onward, (3) studies focused on patients with adult-type glioma with no restrictions on age, (4) studies that used histopathological images gathered from tissue biopsies, tumor excision, or digital histopathological images, (5) studies that assessed the performance of AI algorithms in the detection and classification of the molecular markers, specifically the IDH mutation status and the 1p/19q gene codeletion status using histopathology data, and (6) studies that reported results related to the performance of the developed AI models. No restriction was applied regarding study settings, study design, and country of publication. Studies were excluded if they (1) used data modalities other than histopathology images (eg, radiological images, stimulated Raman histology), (2) did not employ AI algorithms for molecular marker detection or classification, (3) were not human studies, or (4) were categorized as editorials, letters to editors, posters, conference abstracts, reviews, commentaries, short communications, and brief reports.

### Data Extraction

Two authors independently extracted data from the eligible studies using predefined tables in Microsoft Excel sheet. Any discrepancies in the extracted data were resolved through discussion. The extracted data included four main categories: (1) study characteristics, including surname of first author, publication type, publication year, and country of publication; (2) participants and dataset characteristics, including number of participants, mean age, female percentage, dataset size, data source, magnification, slide image resolution, level of analysis, number of classes, and ground truth assessor; (3) AI model characteristics, covering problem-solving approaches, AI classifiers, type of classification, aim of classifier, data input to AI algorithms, features extraction methods, type of validation, and performance metrics; (4) results, including accuracy, sensitivity, specificity, and area under the curve (AUC). The data extraction form is shown in Multimedia Appendix 2.

### Risk of Bias Assessment

To evaluate the quality and reliability of the included studies, two reviewers independently conducted a risk of bias assessment using an adapted version of the QUADAS-2 (Quality Assessment of Diagnostic Accuracy Studies 2) tool [19]. The original QUADAS-2 tool was modified to better suit the context of our review, which focuses on AI applications. Specifically, we incorporated relevant elements from the PROBAST (Prediction Model Risk of Bias Assessment Tool) to ensure that the assessment adequately addressed the methodological nuances of AI-based diagnostic models [20]. Each domain included four tailored signaling questions designed to determine the potential for bias and to evaluate applicability to the review’s research question. The risk of bias was rated separately for each domain, while concerns regarding applicability were assessed for the first three domains. For example, the “Participants” domain examined issues such as sample representativeness, exclusions, and subgroup balance. The “Index Test” domain evaluated the transparency and consistency of the AI model’s design and feature use. The “Reference Standard” focused on the validity and consistency of outcome definitions and assessor qualifications, while the “Analysis” domain addressed data inclusion, preprocessing, set division, and model performance evaluation. In addition to risk of bias, we also assessed concerns regarding applicability in the first three domains, evaluating whether the participants, AI model, and outcome definitions were aligned with the review’s objectives. To refine and validate our modified tool, we conducted a pilot assessment using five studies, which allowed us to fine-tune the criteria before full application. Subsequently, two reviewers independently assessed all included studies using the finalized tool (Multimedia Appendix 3). Any discrepancies in assessments were resolved through discussion and consensus.

### Data Synthesis

A narrative approach was used to synthesize the data extracted from the included studies. Specifically, we used texts and tables to summarize and describe the characteristics of the included studies and results. We could not perform a meta-analysis because fewer than two studies reported the confusion matrices or other necessary details (eg, number of cases and controls in the test set) required to calculate the numerators and denominators for the same performance measures (accuracy, sensitivity, specificity, and AUC), AI algorithms, and measured outcomes (detection of IDH and 1p/19q). Instead of meta-analysis, we calculated the traditional mean for each metric (ie, accuracy, sensitivity, specificity, and AUC).

## Results

### Search Results

**Figure 1.**
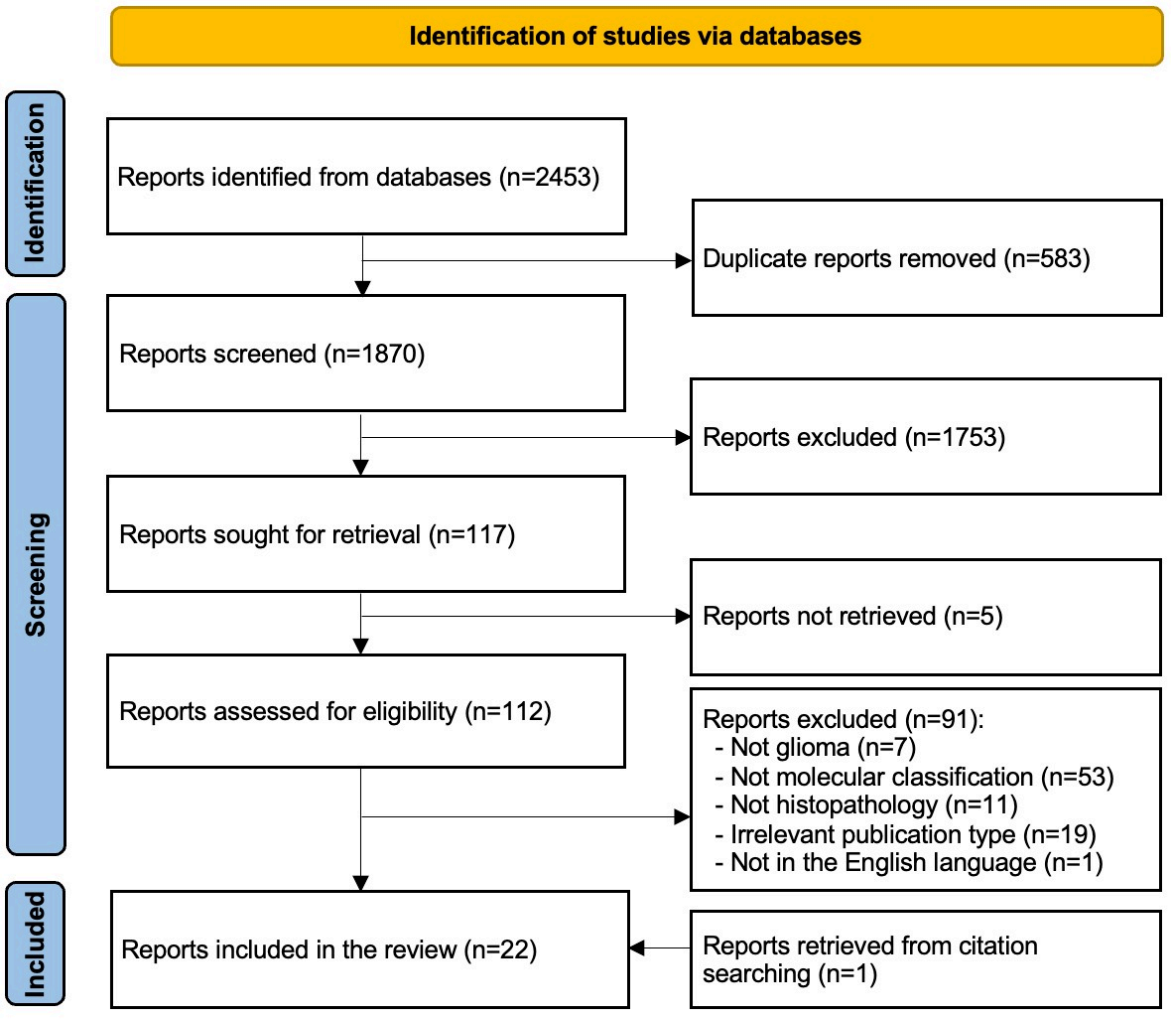
Flowchart of the study selection process.

As shown in Figure 1, the literature searches identified a total of 2453 reports from databases. After removing duplicate records (n=583), we screened the titles and abstracts of 1870 reports. This led to the exclusion of 1753 reports. Of the remaining 117 reports, we could not retrieve the full text for 5 reports. Subsequently, we checked the eligibility of the remaining 112 reports. This led to the exclusion of 91 reports for the following reasons: not related to glioma (n=7), not related to molecular classification (n=53), not pertaining to histopathology (n=11), inappropriate publication type (n=19), and not in the English language (n=1). We included an additional study by checking the reference list of the included studies. Ultimately, only 22 reports met the inclusion criteria and were included in the review [21-42].

### Characteristics of the Included Studies

The included studies were published between 2015 and 2024 (Table 1). The majority of studies were published in 2023 (9/22, 40.91%), followed by 2024 (5/22, 22.73%). Only 3 of the 22 studies (13.64%) are conference papers, whereas 16 (72.73%) are journal articles and 3 (13.64%) are preprints. Studies were carried out in different countries, with the United States and China being the most frequent contributors (6/22, 27.27%) and (5/22, 22.73%), respectively. The number of participants in the included studies ranges from 29 to 2845, with an average of 765.1 (SD 815.6). The mean of female participation rate is 42.45% with a range between 34.5% and 49.3%. The dataset size (ie, number of whole-slide image or patches) varied widely (47‐27,000), with an average of 2464 (SD 5851). It is worth mentioning that the unit of analysis used to compute performance metrics was predominantly whole-slide image level (21/22, 95.5%), while it was at the patch level in 1 (4.5%) study. The participants’ age ranged from 42.2 to 54.5 with an average of 48.03 (SD 4.2; Table 1). The characteristics of each included study are described in Multimedia Appendix 4.

**Table 1.** Characteristics of included studies.

Features	Number of studies	References
Year of publication, n (%)		
2024	5 (22.73)	[24,28,35,37,42]
2023	9 (40.91)	[21,22,25,27,30-33,40]
2022	2 (9.09)	[26,34]
2021	3 (13.64)	[29,38,41]
2020	2 (9.09)	[23,36]
2019	1 (4.55)	[39]
Publication type, n (%)		
Journal article	16 (72.73)	[23,24,26,27,29-31,33-38,40-42]
Conference paper	3 (13.64)	[21,28,32]
Preprint	3 (13.64)	[22,25,39]
Country of publication, n (%)		
United States	6 (27.27)	[28,29,32,34,38,39]
China	5 (22.73)	[23,25,35,40,42]
Germany	2 (9.09)	[21,27]
Australia	2 (9.09)	[36,41]
India	1 (4.55)	[22]
Luxembourg	1 (4.55)	[24]
Canada	1 (4.55)	[26]
Switzerland	1 (4.55)	[30]
South Korea	1 (4.55)	[31]
Saudi Arabia	1 (4.55)	[33]
Japan	1 (4.55)	[37]
Number of participants^a^, mean (SD); range (%)	765.1 (815.6); 29‐2845 (95.45)	[21,22,24-42]
Female percentage^b^ (%), mean (SD); range (%)	42.45 (0.03); 34.5‐49.3 (59.09)	[24,25,29,31,33-37,39-42]
Dataset size^c^, mean (SD); range (%)	2464 (5851); 47‐27,000 (95.45)	[22-42]
Mean age^d^ (year), mean (SD); range (%)	48.03 (4.2); 42.2‐54.5 (59.09)	[24,25,29,31,33-37,39-42]

a One study did not report the mean age [23].

b Studies did not report the female percentage [21-23,26-28,30,32,38].

c One study did not report the dataset size [21].

d Studies did not report the mean age [21-23,26-28,30,32,38].

### Features of Histopathological Images

As presented in Table 2, the histopathological images were captured at various magnification levels, including 2.5×, 5×, 10×, 20×, 40×, and 100×. Among these, 20× magnification was the most commonly used (12/22, 54.55%). While 14 (63.64%) studies focused on architectural-level analysis, 13 (59.09%) studies focused on cellular-level analysis. Slide resolutions varied across studies, with 256×256 pixels being the most common (8/22, 36.36%). Datasets were sourced from either open-source databases (16/22, 72.73%) or closed-source datasets (13/22, 59.09%) (ie, data were collected by the study authors or obtained from previous studies). The open datasets used include The Cancer Genome Atlas (15/22, 68.18%) and the Digital Brain Tumor Atlas (1/22, 4.55%), with some studies combining open and closed sources. The histopathological images were labeled into 2 classes in about two-thirds of the studies (14/22, 63.64%). The features of histopathological images in each study are described in Multimedia Appendix 5.

**Table 2.** Features of histopathological images^a^.

Features	Number of studies, n (%)	References
Magnification		
2.5×	1 (4.55)	[34]
5×	2 (9.09)	[32,34]
10×	4 (18.18)	[24,29,34,36]
*20×^b^*	*12* (54.55)	[22,24-26,28,31,32,34,35,40-42]
40×	5 (22.73)	[24,30,31,35,41]
100×	1 (4.55)	[24]
Not reported	7 (31.82)	[21,23,27,33,37-39]
Level of analysis		
*Architecture level*	*14* (63.64)	[22,24-26,28,29,31,32,34-36,40-42]
Cell level	13 (59.09)	[22,24-26,28,30-32,34,35,40-42]
Not reported	7 (31.82)	[21,23,27,33,37-39]
Slide image resolution		
224×224	4 (18.18)	[29,31,37,42]
*256×256*	*8* (36.36)	[22,23,25,28,32,34,36,37]
512×512	3 (13.64)	[21,23,38]
1024×1024	5 (22.73)	[23,33,35,36,40]
Other	7 (31.82)	[21,23,24,26,30,41,42]
Not reported	2 (9.09)	[27,39]
Data sources		
*Open source*	*16* (72.73)	[21-23,25,27-29,31-34,36-39,41]
Closed source	13 (59.09)	[24-27,30,31,33-36,40-42]
Open dataset name		
** ** *TCGA* ^c^	*15* (68.18)	[21,23,25,27-29,31-34,36-39,41]
DBTA^d^	1 (4.55)	[22]
Not applicable	6 (27.27)	[24,26,30,35,40,42]
Number of classes		
*Two*	*14* (63.64)	[22,23,28-37,41,42]
Three	2 (9.09)	[21,26]
Four	4 (18.18)	[25,27,38,39]
Five	2 (9.09)	[24,33]
Six	1 (4.55)	[40]

a Studies used both data sources, open and closed [25,31,33,34,36,41].

b Values in italics indicate highest score.

c TCGA: The Cancer Genome Atlas.

d DBTA: Digital Brain Tumor Atlas.

### Features of AI

AI was primarily used for binary classification in 86.36% (19/22) of studies, where models differentiate between two outcomes (eg, IDH-mutant vs wild-type), whereas only 22.73% (5/22) employed multiclass classification, which predicts among 3 or more glioma subtypes (Table 3). The majority of studies (20/22, 90.91%) used AI for IDH subtyping (ie, distinguishing between IDH mutant and IDH wild-type), whereas only 6 (27.27%) studies applied AI for subtyping IDH mutant tumors in relation to 1p/19q gene codeletion. Convolutional neural networks (CNNs) were the most commonly used classifier and feature extraction method, appearing in 54.55% (12/22) and 90.91% (20/22) of studies. For specific classifiers, CNNs are the most utilized, appearing in 54.55% (12/22) of studies, followed by multiple instance learning (MIL) (which handles weakly labeled slide-level data by learning from sets of image patches without requiring pixel-level annotations) at 50% (11/22), transformers at 18.18% (4/22), and hybrid models (ie, single end-to-end architectures that integrate convolutional components with attention-based mechanisms [eg, CNN-transformer models] within a unified computational graph and are trained jointly) also at 18.18% (4/22). For specific feature extraction models, ResNet50 leads with 46.67% (10/22) usage, followed by densely connected convolutional network 121 (DenseNet121) and InceptionV3, both at 19.05% (4/22). Among the included studies, most models were developed using histopathological images alone (17/22, 77.3%), while a smaller number (5/22, 22.7%) incorporated additional modalities such as MRI scans (2/22, 9.1%), demographic information (3/22, 13.6%), and clinical variables (2/22, 9.1%). The most widely used method for model validation was k-fold cross-validation, used in 50% (11/22) of studies, followed by the train-test split approach in 45.45% (10/22) of studies. AI model performance was assessed using multiple metrics, with accuracy being the most frequently reported (19/22, 86.36%), followed by AUC and sensitivity, each used in 68.18% (15/22) of studies. A detailed summary of the AI models used in each study is provided in Multimedia Appendix 6.

**Table 3.** Features of artificial intelligence (AI).

Feature	Number of studies, n (%)	References
Type of classification		
*Binary^a^*	*19* (86.36)	[22,23,25,27-42]
Multiclass	5 (22.73)	[21,24,26,33,40]
Aim of AI algorithms^b^		
*Subtyping IDH*^c^	*20* (90.91)	[21-29,32-42]
Subtyping 1p/19q codeletion	6 (27.27)	[25,27,30,31,38,39]
Classifier		
*Convolutional neural networks*	*12* (54.55)	[24,26,29,30,33-36,38-40,42]
Multiple instance learning	11 (50)	[21-23,25,27,28,31-33,37,42]
Transformers	4 (18.18)	[30,33,35,42]
Hybrid models	2 (9.09)	[35,42]
Ensemble model	2 (9.09)	[34,37]
Logistic regression	2 (9.09)	[31,36]
Random forest	1 (4.55)	[41]
Multilayer perceptron	1 (4.55)	[21]
Specific classifier		
*AttMIL*^d^	*7* (31.82)	[22,23,25,27,28,33,37]
ResNet50	5 (22.73)	[24,35,36,40,42]
Traditional MIL^e^	4 (18.18)	[21,31,33,42]
InceptionV3	4 (18.18)	[24,33,35,36]
ViT^f^	4 (18.18)	[24,33,35,42]
DenseNet121^g^	3 (13.64)	[24,34,35]
VGG19^h^	3 (13.64)	[24,26,36]
Features extraction methods		
*Convolutional neural networks*	*20* (90.91)	[21-26,28-40,42]
Transformers	4 (18.18)	[33,35,37,42]
Hybrid models	4 (18.18)	[27,35,37,42]
MIL	1 (4.55)	[42]
Random forest	1 (4.55)	[41]
Specific features extraction model		
*ResNet50*	*10* (45.45)	[22,24,25,28,31,35-37,40,42]
DenseNet121	5 (22.73)	[21,22,24,34,35]
InceptionV3	4 (18.18)	[24,33,36,37]
ResNet18	3 (13.64)	[24,29,32]
VGG19	2 (9.09)	[24,26,36]
ViT	2 (9.09)	[33,42]
Not reported	2 (9.09)	[23,30]
Data modality		
Unimodel data	17 (77.3)	[22-35,39,40,42]
Multimodel data	5 (22.7)	[21,36-38,41]
Types of validation		
*k-fold cross-validation*	*11* (50)	[21,23,25,27,28,31,33,37-39,41]
Training-test split	10 (45.45)	[22,24,26,29,30,32,34-36,40]
External validation	7 (31.82)	[25,27,29,31,34,40,42]
Performance measures		
*Accuracy*	*19* (86.36)	[21-25,27,28,31-42]
AUC^i^	15 (68.18)	[21-23,26,28,29,31,33-36,39-42]
Sensitivity	15 (68.18)	[23-25,27,28,30-32,34-37,39,40,42]
*F*_1_-score	9 (40.91)	[21,24,25,31,32,35,37,40,42]
Precision	8 (36.36)	[23,24,27,30-32,35,37]
Specificity	8 (36.36)	[25,27,28,34,36,39,40,42]
Others	8 (36.36)	[21,22,25,27-29,37,39]

a Values in italic indicate highest score.

b Studies reported results for both IDH mutation status and 1p/19q codeletion status [25,27,38,39].

c IDH: isocitrate dehydrogenase.

d AttMIL: attention-based multiple instance learning.

e MIL: multiple instance learning.

f ViT: vision transformer.

g DenseNet121: densely connected convolutional network 121.

h VGG: visual geometry group.

i AUC: area under the curve.

### Results of the Risk of Bias and Applicability

**Figure 2.**
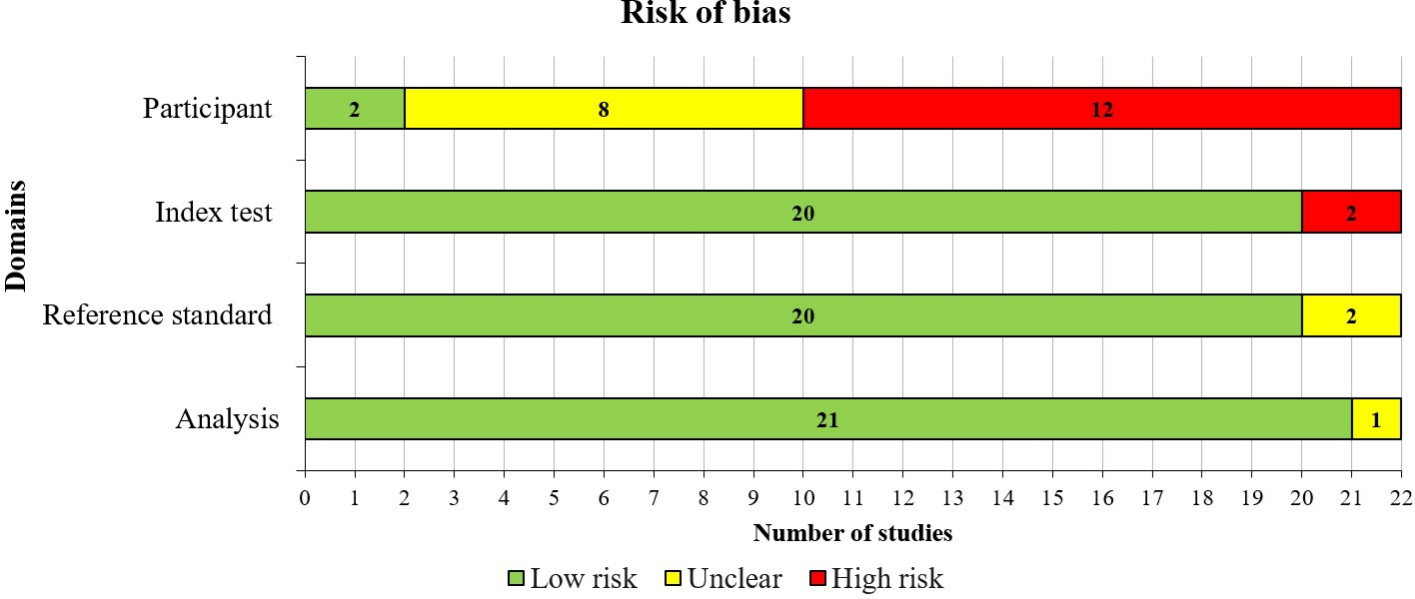
Results of the assessment of risk of bias in the included studies.

In the selection of participants domain, nearly a quarter of the included studies (5/22, 23%) provided sufficient information to confirm the use of an appropriate consecutive or random sampling of eligible participants. Nearly all studies (21/22, 95%) reported a sufficient sample size. Additionally, 8 (36%) studies avoided inappropriate exclusions, while over half (12/22, 55%) demonstrated balanced subgroup representation. Consequently, a small proportion of the studies (2/22, 9%) were assessed as having a low risk of bias, while a larger portion (8/22, 36%) were rated as having an unclear risk of bias in the “selection of participants” domain, as shown in Figure 2.

**Figure 3.**
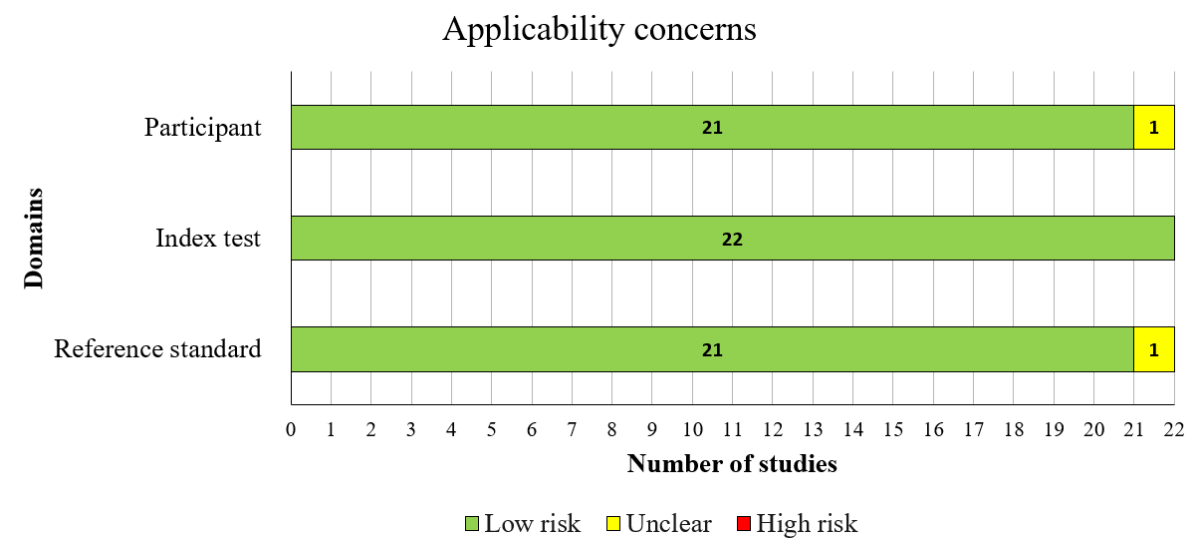
Results of the assessment of applicability concerns in the included studies.

In terms of matching participants to the predefined requirements of the review question, a low level of concern was identified in 95% (21/22) of the included studies, as shown in Figure 3.

A substantial majority of the included studies comprehensively detailed their AI models, with 21 of the 22 (95%) studies providing thorough descriptions. An overwhelming majority, all 22 studies (100%) clearly reported the features (predictors) used. Moreover, all 22 studies (100%) ensured that these features were sourced without prior knowledge of the outcome data. Consistency in feature assessment across participants was observed in 21 of the 22 (95%) studies. Consequently, the potential for bias in the “index test” domain was assessed as low in 20 of the 22 (91%) studies**,** as shown in Figure 2. In addition, all 22 (100%) studies were found to have minimal concerns regarding the alignment between the model’s predictors and the review question’s criteria, as illustrated in Figure 3.

In most of the included studies (20/22, 91%), the outcome of interest, histopathology image analysis, was conducted by an expert (eg, pathologist or neuropathologist), ensuring the necessary expertise to accurately classify the outcomes. Nearly all studies (22/22, 100%) applied consistent and uniform outcome definitions across all participants, with outcome classification (ie, image labeling) blinded to the AI model predictions. An overwhelming majority (22/22, 100%) determined the outcome without prior knowledge of the predictor information. Furthermore, in all 22 (100%) studies, the diagnostic process was carried out over an appropriate duration, using the same diagnostic criteria for all participants. As a result, the risk of bias in the “reference standard” domain was considered low in the majority of studies (20/22, 91%), as shown in Figure 2. Additionally, a similar proportion of studies (21/22, 95%) exhibited minimal concerns regarding discrepancies in outcome definition, timing, and determination methods, as shown in Figure 3.

Finally, a significant majority of the studies (22/22, 100%) ensured the inclusion of all enrolled participants in the data analysis. A substantial number of these studies (21/22, 95%) reported no missing values, or if missing values were present, they were handled appropriately (eg, through multiple imputation).

Similarly, a high proportion of studies (22/22, 100%) adopted suitable measures to evaluate the performance of their models. The confusion matrix was presented, or more than one evaluation measure was used, and the selected measures were deemed appropriate.

Nearly half of the studies (21/22, 95%) demonstrated an appropriate split among training, validation, and test sets. The chosen distribution aligned with best practices in the field (eg, 70%‐80% for training, 10%‐15% for validation, and 10%‐20% for testing).

Consequently, the conduct and interpretation of the analysis did not introduce bias in most studies, with 21/22 (95%) having a low risk of bias in the “analysis” domain, as shown in Figure 2. A detailed breakdown of the “risk of bias” and “applicability concerns” for each domain in every study is available in Multimedia Appendix 7.

### Results of the Included Studies

As shown in Table 4, the accuracy of AI models in detecting or subtyping molecular markers in adult-type gliomas ranged from 58% to 100%, with a mean accuracy of 85.46%. Sensitivity ranged from 62% to 100%, averaging 84.55%, while specificity ranged from 46% to 100%, with a mean of 86.03%. The AUC values ranged from 46% to 99%, with an average of 86.53%. The hybrid model demonstrated the highest accuracy (92.80%) and sensitivity (89.62%), while the CNN model achieved the highest specificity (89.30%). Logistic regression outperformed other models in terms of AUC (93.05%). Among specific CNN architectures, DenseNet121 achieved the highest accuracy (90.82%), sensitivity (89.28%), and AUC (91.05%). Moreover, AI models that used multimodal data outperformed those that used unimodal data in terms of sensitivity (90.15% vs 84.31%) and AUC (88.93% vs 86.29%). In outcome prediction, AI models showed better performance in detecting IDH mutations compared to 1p/19q codeletions, with higher accuracy (86.13% vs 81.63%), specificity (86.61% vs 78.11%), and AUC (86.74% vs 85.15%). AI models also performed better in IDH subtyping for multiclass classification compared to binary classification in terms of accuracy (91.98% vs 84.02%) and sensitivity (93.41% vs 80.18%). Conversely, AI models for binary classification yielded a lower performance than multiclass classification in terms of accuracy (91.98% vs 84.02%) and sensitivity (93.41% vs 80.18%). However, these differences should be interpreted as descriptive trends rather than statistically validated superiority, as formal between-group comparisons were not feasible.

**Table 4.** Summary of overall performance of all models based on number of classes, type of data, and artificial intelligence (AI) algorithms in the target variable^a^.

Groups	Accuracy			Sensitivity			Specificity			AUC^b^		
	Number of studies	Mean % (SD)	Range	Number of studies	Mean % (SD)	Range	Number of studies	Mean % (SD)	Range	Number of studies	Mean % (SD)	Range
Algorithms												
*CNNs***^c^**	8	85.60 (0.09)	0.72‐1.00	8	84.09 (0.11)	0.64‐1.00	7	*89.30*^d^ (0.09)	0.75‐1.00	8	86.54 (0.06)	0.76‐0.99
MIL^e^	10	80.83 (0.09)	0.58‐0.91	8	82.54 (0.13)	0.62‐0.99	3	68.69 (0.23)	0.46‐0.94	10	81.73 (0.16)	0.46‐0.97
Transformer	4	88.27 (0.03)	0.83‐0.92	3	81.62 (0.10)	0.69‐0.92	—	—	—	3	92.37 (0.04)	0.89‐0.96
*Hybrid model*	3	*92.80* (0.05)	0.85‐0.97	3	*89.62* (0.08)	0.81‐0.97	—	—	—	2	92.20 (0.04)	0.89‐0.95
Ensemble model	2	86.35 (0.12)	0.78‐0.95	2	89.60 (0.05)	0.86‐0.93	—	—	—	2	92.00 (0.09)	0.85‐0.99
*Logistic regression*	2	87.15 (0.01)	0.86‐0.88	2	86.00 (0.12)	0.78‐0.94	—	—	—	2	*93.05* (0.00)	0.93‐0.931
CNN models												
*ResNet (ResNet,18,50)*	7	87.04 (0.09)	0.72‐1.00	6	87.51 (0.11)	0.69‐1.00	5	*91.48* (0.07)	0.84‐1.00	6	90.19 (0.06)	0.81‐0.99
VGG^f^ (VGG16,19)	4	89.67 (0.09)	0.79‐1.00	4	87.03 (0.13)	0.69‐1.00	2	90.65 (0.13)	0.81‐1.00	2	81.55 (0.01)	0.81‐0.82
*DenseNet121***^g^**	3	*90.82* (0.10)	0.79‐0.97	3	*89.28* (0.10)	0.78‐0.97	—	—	—	2	*91.05* (0.08)	0.86‐0.97
InceptionV3	3	80.15 (0.05)	0.77‐0.86	3	75.82 (0.11)	0.64‐0.86	—-	—	—	3	83.58 (0.03)	0.80‐0.87
Type of data												
Unimodel data	20	*85.67* (0.09)	0.58‐1.00	16	84.31 (0.11)	0.62‐1.00	9	*86.27* (0.15)	0.46‐1.00	16	86.29 (0.10)	0.46‐0.99
Multimodel data	5	83.64 (0.05)	0.78‐0.90	2	*90.15* (0.06)	0.86‐0.94	—	—	—	4	*88.93* (0.03)	0.85‐0.93
Outcome prediction												
1p/19q	6	81.63 (0.12)	0.58‐1.00	6	*87.64* (0.09)	0.75‐1.00	3	78.11 (0.28)	0.46‐1.00	5	85.15 (0.19)	0.46‐0.99
IDH^h^	19	*86.13* (0.08)	0.72‐1.00	14	83.96 (0.11)	0.62‐1.00	8	*86.61* (0.12)	0.52‐1.00	16	*86.74* (0.08)	0.52‐0.99
Class prediction												
Binary IDH	16	84.02 (0.07)	0.72‐1.00	12	80.18 (0.11)	0.62‐1.00	8	*86.61* (0.12)	0.52‐1.00	15	*86.87* (0.08)	0.52‐0.99
Multiclass IDH	3	*91.98* (0.07)	0.75‐1.00	2	*93.41* (0.05)	0.85‐1.00	—	—	—	—	—	—
*Overall*	*20*	*85.46* (0.08)	*0.58‐1.00*	*16*	*84.55* (0.01)	*0.62‐1.00*	*9*	*86.03* (0.15)	*0.46‐1.00*	*17*	*86.53* (0.10)	*0.46‐0.99*

a Specificity not reported in all studies.

b AUC: area under the curve.

c CNNs: convolutional neural networks.

d Values in italics indicate highest score.

e MIL: multiple instance learning.

f VGG: visual geometry group.

g DenseNet121: densely connected convolutional network 121.

h IDH: isocitrate dehydrogenase.

## Discussion

### Principal Findings

This review aimed to systematically evaluate the performance of AI models in detecting and classifying IDH mutation status and 1p/19q codeletion in adult-type gliomas using histopathological images. Our analysis indicates that these models demonstrate promising diagnostic capabilities, achieving pooled mean values of 85.46% for accuracy, 84.55% for sensitivity, 86.03% for specificity, and 86.53% for the area under the receiver operating characteristic curve. As noticed, the performance across all metrics was comparable, suggesting that AI models offer balanced diagnostic capabilities. Despite these promising results, they remain below the commonly cited thresholds for clinical adoption, which often exceed 90% for high-stakes diagnostic tasks [42]. As such, the current evidence is not sufficient to support stand-alone clinical decision-making. Therefore, the results of this meta-analysis should be interpreted as early indicators of feasibility rather than clinically actionable performance, underscoring the substantial methodological and validation work still required before these models can be safely integrated into clinical workflows.

These results are generally consistent with prior reviews. Specifically, a systematic review conducted by Farahani et al [43] reported a similar performance in IDH prediction using MRI, with pooled sensitivity of 84% and specificity of 87% and an AUC of 0.89. Further, Chen et al [44] found pooled sensitivity of 83%, specificity of 84%, and AUC of 0.90 for predicting IDH mutations using machine learning–based radiomics. Taken together, these comparisons suggest that our findings are in line with the broader body of research, reinforcing the reliability and clinical promise of AI-based histopathological approaches for glioma molecular classification.

Individual studies showed considerable variability across performance metrics; for instance, specificity values ranged from 46% to 100%. This variability likely stems from population differences, sample sizes, model architectures, and the inherent challenge of predicting rare molecular alterations (eg, telomerase reverse transcriptase mutations) from histopathology images, compounded by class imbalance, domain shift (ie, performance degradation when models are applied to data from different sources), and the absence of distinct histological correlates. While accuracy and sensitivity remain higher for well-characterized targets (eg, IDH, 1p/19q), specificity suffers when the model encounters rare or ambiguous cases in external validation.

Hybrid models achieved the highest mean accuracy (92.80%) and sensitivity (89.62%), while logistic regression models achieved the top mean AUC just slightly higher than hybrid models (93.05% vs 92.20%). These results suggest that hybrid models deliver the strongest overall performance. Hybrid models are defined as AI frameworks that integrate multiple deep learning architectures to leverage the complementary strengths of each component. In the context of glioma diagnosis, most recent reviews have highlighted the effectiveness of hybrid models combining CNNs with transformer-based architectures [45-47]. CNNs excel at extracting local spatial features from histopathological images, such as cellular morphology and texture patterns, while transformers are adept at capturing long-range dependencies and global contextual information through their self-attention mechanisms. By combining these capabilities, hybrid CNN-transformer models can achieve a more comprehensive understanding of complex tissue structures and molecular characteristics within gliomas, leading to improved predictive performance. Our findings align with a recent review by Redlich et al [45], which analyzed 83 studies on AI-based methods for whole-slide histopathology image analysis of gliomas. The review highlighted that hybrid models combining CNNs with other architectures, including transformers, consistently outperform single-architecture models in tasks such as subtyping, grading, and molecular marker prediction [45]

Among pure CNN backbones, DenseNet121 demonstrated the best performance, achieving an accuracy of 90.82%, sensitivity of 89.28%, and an AUC of 91.05%. DenseNet121’s superior performance may be due to its unique architectural features, notably dense connectivity, which enhance training dynamics, feature representation, and parameter efficiency.

In terms of data modalities employed by AI models, our analysis demonstrated that multimodal approaches exhibited superior performance in sensitivity (90.15% vs 84.31%) and AUC (88.93% vs 86.29%) compared to unimodal models. This suggests that multimodal models benefit from richer and more diverse feature representations, thereby enhancing their diagnostic capability. However, unimodal models achieved slightly higher accuracy (85.67% vs 83.64%), which may be attributed to the fact that none of the five multimodal studies included in the accuracy analysis employed hybrid modeling. Instead, they utilized simple model ensembles or comparisons involving architectures such as multilayer perceptron, logistic regression, random forests, and CNNs. Our findings are consistent with existing literature. For instance, a systematic review by Alleman et al [48] concluded that multimodal deep learning approaches enhance prognostic accuracy in glioma patients compared to unimodal models, particularly in predicting overall survival. Similarly, d’Este et al [49] demonstrated that combining multimodal imaging with AI techniques improves visualization of glioma infiltration, surpassing the capabilities of single-modality approaches. Furthermore, the GlioMT model, introduced by Byeon et al [50], which integrates imaging and clinical data, exhibited superior performance in predicting adult-type diffuse gliomas compared to conventional CNNs and visual transformers.

Regarding outcome prediction and AI model performance in detecting molecular markers, our analysis revealed that AI models demonstrated stronger performance in identifying IDH mutations compared to 1p/19q codeletions. Specifically, IDH detection yielded higher accuracy (86.13% vs 81.63%), specificity (86.61% vs 78.11%), and AUC (86.74% vs 85.15%). In contrast, AI models achieved slightly better sensitivity for 1p/19q codeletion detection (87.64% vs 83.96%). The discrepancy in performance between IDH mutation and 1p/19q codeletion detection may be attributed to several factors. IDH mutations often result in more distinct histopathological features, making them more amenable to detection by AI models. In contrast, 1p/19q codeletions may not produce as pronounced morphological changes, posing a greater challenge for AI-based detection. The findings of published studies align with our results. For example, the review by Farahani et al [43] showed higher performance in identifying IDH mutations compared to 1p/19q codeletions using MRI in terms of sensitivity (84% vs 76%) and specificity (87% vs 85%).

In our systematic review, CNNs and MIL algorithms were the most predominantly used in AI models, likely due to their suitability for histopathological image analysis. CNNs are designed to capture spatial hierarchies in image data through convolutional layers, making them effective at identifying morphological patterns such as cellular structures and tissue architecture that are critical in tumor classification. Histopathological slides of gliomas are typically of high resolution and contain complex visual features, which CNNs can learn to recognize with high accuracy. Meanwhile, MIL frameworks address the challenge of weakly labeled data, which is common in medical imaging, where only slide-level labels (eg, tumor type or mutation status) are available, rather than precise annotations at the pixel or region level. MIL allows models to learn from sets of image patches (instances) while associating predictions with the whole slide (bag), making it particularly useful in computational pathology where exhaustive labeling is impractical.

### Practical and Research Implications

While our results are encouraging, they remain not optimal and arguably lower than that acceptable in clinical practice [42]. Thus, our findings should be interpreted with caution due to several limitations. First, 15 of the 22 studies included in the review utilized the same dataset, raising concerns regarding the generalizability of the results. Second, many of the studies were based on small sample sizes, limiting the robustness and reliability of the conclusions. Third, several key findings were derived from fewer than 5 studies, reducing the strength of the evidence. Lastly, the aggregation of results using simple arithmetic means rather than formal meta-analytic techniques may have limited the ability to draw definitive conclusions about the overall efficacy of AI models.

Given these limitations, AI should not be used as a stand-alone diagnostic tool for the detection or subtyping of adult-type gliomas but rather as a complementary approach alongside conventional methods. Future research should prioritize the development of AI models using larger and more diverse datasets to enhance performance and generalizability. Ensuring adequate sample sizes and improving the transparency of reporting, such as including confusion matrices, will also facilitate more rigorous meta-analyses. Notably, the majority of studies reviewed developed AI models exclusively from histopathological images. Developing multimodal AI models that incorporate additional data types (eg, radiological and clinical information) may further enhance diagnostic accuracy. Thus, continued efforts are needed to advance the integration of multimodal data in AI model development for the detection and subtyping of adult-type gliomas.

In this review, the majority of studies used AI algorithms that were either MIL or CNNs, indicating a research gap in exploring the performance and effectiveness of other types of AI algorithms (eg, transformers, hybrid models, ensemble models [ie, approaches that combine predictions from multiple independently trained models using averaging, voting, or weighted fusion strategies], or logistic regression). Additionally, we observed a lack of research assessing the performance of AI models that use multimodal data, which may have affected the accuracy of the results. Moreover, the number of studies evaluating AI models’ performance in detecting IDH mutations was noticeably greater than those that used 1p/19q as a predicted outcome. These studies predominantly employed binary IDH classification (ie, IDH mutation vs IDH wild-type), whereas studies using IDH multiclass classification (eg, astrocytoma, oligodendroglioma) were markedly fewer. Future research should address these gaps to produce more accurate and representative findings.

Our findings that AI hybrid models performed better in detecting adult-type gliomas than other models underscore the need for more advanced approaches to explore their effectiveness and their potential to achieve diagnostic outcomes comparable to those of histopathologists. However, their real-world applicability requires consideration of computational and operational demands. These models often require greater processing power (eg, GPU-enabled servers or high-performance cloud computing) and longer inference times compared to conventional CNNs. Such requirements may pose challenges for pathology laboratories lacking advanced digital infrastructure. Nonetheless, many modern clinical centers are increasingly equipped with GPU-accelerated digital pathology ecosystems, and cloud-based deployment can significantly reduce the need for on-site hardware. From a cost-benefit perspective, the additional computational resources required by hybrid models may be justified given their superior accuracy and robustness, particularly for molecular prediction tasks where diagnostic errors carry serious clinical implications. However, successful implementation in routine practice will depend on streamlined model optimization, efficient inference pipelines, and prospective validation of runtime performance to ensure that diagnostic gains meaningfully outweigh operational costs.

As our findings indicated superior performance of multimodal AI models in sensitivity and AUC, we suggest a complementary approach: multimodal AI models may serve as effective initial diagnostic tools due to their comprehensive data integration, while unimodal models can be employed subsequently for more accurate and specific outcome determination. Moreover, our results reflect a stronger performance of AI models in the detection of IDH mutations compared to 1p/19q codeletion in all metrics except sensitivity. Therefore, we suggest that while IDH mutation detection should remain a primary focus for model refinement and deployment due to its balanced high performance, models targeting 1p/19q codeletions may serve as effective initial diagnostic tools in adult-type gliomas due to their higher sensitivity, which is critical in early disease detection.

Given that the current review focused on IDH mutations and 1p/19q codeletions, we encourage researchers to undertake further reviews covering other glioma molecular markers, including MGMT methylation, ATRX, and telomerase reverse transcriptase mutations. Moreover, further research should extend to other types of brain tumors, such as meningiomas, pituitary adenomas, and schwannomas. These tumors differ significantly in terms of biological behavior, treatment options, and clinical outcomes, warranting a comprehensive evaluation of AI’s potential in diagnosing and subtyping each tumor type.

### Limitations

This systematic review is subject to several limitations. First, the generalizability of the findings is limited, as approximately 68.18% of the included studies relied on the same dataset, and the number of included studies is relatively small (n=22). This raises concerns about the external validity of the results and their applicability to the broader population of adult-type glioma patients. To address the potential duplication of patients or slides across studies (ie, those relying on The Cancer Genome Atlas), we reviewed the data sources reported by each study to identify where overlap was likely. Because individual-level data were not available, we were unable to verify or remove duplicate cases. Instead, we treated studies drawing from the same dataset as nonindependent samples and interpreted pooled estimates with caution. As a result, our summary metrics should be viewed as descriptive indicators rather than independent, unbiased effect estimates. Second, the review focused exclusively on adult-type gliomas, thereby restricting the ability to draw conclusions about the performance of AI in detecting other adult brain tumors or brain tumors in the general population. Third, the analysis was confined to studies utilizing histopathological images, limiting the assessment of AI performance with alternative imaging modalities, such as MRI or computed tomography scans. Fourth, meta-analysis was not feasible due to incomplete reporting of key performance metrics across studies. Instead, mean values were calculated to provide a descriptive summary, though this approach lacks the statistical rigor and precision of formal meta-analysis. Finally, the inclusion of only English-language studies may have introduced language bias and led to the exclusion of relevant research published in other languages, potentially affecting the comprehensiveness of the review.

### Conclusion

This systematic review demonstrates that AI models applied to histopathological images show strong potential for the molecular classification of adult-type gliomas, particularly in detecting IDH mutations and 1p/19q codeletions. Our findings indicate that while overall diagnostic performance is promising—especially for IDH mutations—variability in model performance and methodological limitations across studies temper the generalizability of results. Hybrid models and multimodal approaches emerged as particularly effective strategies, offering enhanced sensitivity and discriminative capability. However, the limited use of diverse datasets, the predominance of unimodal designs, and the lack of standardized reporting remain critical barriers to clinical translation. Given these constraints, AI models should currently serve as complementary tools rather than stand-alone diagnostic systems, functioning primarily to assist pathologists in pattern recognition and decision support while final diagnoses remain clinician-led. Future research should prioritize methodological standardization, broader data integration, and validation across larger, heterogeneous populations to support the clinical deployment of AI in glioma diagnostics.

## Supplementary material

10.2196/78377 Multimedia Appendix 1 Search strategy.

10.2196/78377 Multimedia Appendix 2 Data extraction form.

10.2196/78377 Multimedia Appendix 3 Modified version of QUADAS-2.

10.2196/78377 Multimedia Appendix 4 Characteristics of each included study.

10.2196/78377 Multimedia Appendix 5 Features of histopathological images in each study.

10.2196/78377 Multimedia Appendix 6 Features of artificial intelligence in each study.

10.2196/78377 Multimedia Appendix 7 Reviewers’ judgments about each domain in “risk of bias” and "applicability concerns" for each included study.

10.2196/78377 Checklist 1 PRISMA-DTA checklist.

## Abbreviations

AI: artificial intelligence
AUC: area under the curve
CNN: convolutional neural network
CNS: central nervous system
DBTA: Digital Brain Tumor Atlas
DenseNet121: densely connected convolutional network 121
IDH: isocitrate dehydrogenase
MIL: multiple instance learning
MRI: magnetic resonance imaging
PRISMA-DTA: Preferred Reporting Items for Systematic Reviews and Meta-Analyses–Extension for Diagnostic Test Accuracy
PROBAST: Prediction Model Risk of Bias Assessment Tool
PROSPERO: International Prospective Register of Systematic Reviews
QUADAS-2: Quality Assessment of Diagnostic Accuracy Studies 2
WHO: World Health Organization
