# Supplementary material for: The Performance of Artificial Intelligence in Classifying Molecular Markers in Adult-Type Gliomas Using Histopathological Images: Systematic Review
Source: J Med Internet Res. 2026 Mar 13;28:e78377. doi: 10.2196/78377 (PMC12986776; doi:10.2196/78377)
Supplement: Multimedia Appendix 1 [file jmir-v28-e78377-s001.docx]

**Multimedia Appendix 2: Search Strategy**

Database(s): **Ovid MEDLINE(R) ALL**1946 to November 15, 2024
Search Strategy:

| **#** | **Searches** | **Results** |
| --- | --- | --- |
| 1 | exp astrocytoma/ or exp diffuse intrinsic pontine glioma/ or exp ependymoma/ or exp gliosarcoma/ or exp oligodendroglioma/ | 54617 |
| 2 | Glioma*.tw. | 75356 |
| 3 | "isocitrate dehydrogenase mutation*" | 271 |
| 4 | "isocitrate dehydrogenase 1 mutation*" | 107 |
| 5 | "isocitrate dehydrogenase 2 mutation*" | 14 |
| 6 | "chromosome 1p and 19q codeletion*" | 1 |
| 7 | "1p/19q codeletion" | 577 |
| 8 | Astrocytoma*.tw. | 18017 |
| 9 | Glioblastoma*.tw. | 53346 |
| 10 | Gliosarcoma*.tw. | 1263 |
| 11 | ependymoma*.tw. | 6210 |
| 12 | oligodendroglioma*.tw. | 4789 |
| 13 | astroblastoma*.tw. | 234 |
| 14 | Xanthoastrocytoma*.tw. | 703 |
| 15 | "Glial cell tumor*".tw. | 58 |
| 16 | "Glial tumor*".tw. | 2152 |
| 17 | exp Artificial Intelligence/ | 214578 |
| 18 | "Artificial Intelligence".tw. | 51304 |
| 19 | "Machine Learning".tw. | 113577 |
| 20 | "Deep Learning".tw. | 63908 |
| 21 | "Generative Pre-trained Transformer".tw. | 493 |
| 22 | "Generative Pre trained Transformer*".tw. | 538 |
| 23 | "Generative AI".tw. | 666 |
| 24 | "Large language model*".tw. | 3124 |
| 25 | "pre-trained transformer".tw. | 556 |
| 26 | "Decision Tree*".tw. | 17636 |
| 27 | "K-Nearest Neighbor*".tw. | 6380 |
| 28 | "Support vector machine*".tw. | 29796 |
| 29 | "Recurrent Neural Network*".tw. | 4876 |
| 30 | "Convolutional Neural Network*".tw. | 29733 |
| 31 | "Artificial neural network*".tw. | 19557 |
| 32 | "Deep Neural Network*".tw. | 11394 |
| 33 | "Naïve Bayes".tw. | 9 |
| 34 | "Naive Bayes".tw. | 3880 |
| 35 | "Bayesian Networks".tw. | 1651 |
| 36 | "Fuzzy Logic".tw. | 2662 |
| 37 | "K-Means".tw. | 8431 |
| 38 | "Random Forest*".tw. | 30094 |
| 39 | "Long Short-Term Memory".tw. | 5916 |
| 40 | "Gradient Boost*".tw. | 8443 |
| 41 | AdaBoost.tw. | 1759 |
| 42 | "Multilayer Perceptron".tw. | 3280 |
| 43 | "Ensemble learning".tw. | 2395 |
| 44 | "Generative Adversarial Network*".tw. | 3991 |
| 45 | "Transfer Learning".tw. | 6449 |
| 46 | histopatholog*.tw. | 300330 |
| 47 | patholog*.tw. | 1036913 |
| 48 | histolog*.tw. | 663073 |
| 49 | "whole-slide image*".tw. | 2235 |
| 50 | Immunohistochemistry.tw. | 232311 |
| 51 | 1 or 2 or 3 or 4 or 5 or 6 or 7 or 8 or 9 or 10 or 11 or 12 or 13 or 14 or 15 or 16 | 133951 |
| 52 | 17 or 18 or 19 or 20 or 21 or 22 or 23 or 24 or 25 or 26 or 27 or 28 or 29 or 30 or 31 or 32 or 33 or 34 or 35 or 36 or 37 or 38 or 39 or 40 or 41 or 42 or 43 or 44 or 45 | 384558 |
| 53 | 46 or 47 or 48 or 49 or 50 | 1979450 |
| 54 | 51 and 52 and 53 | 610 |
| 55 | limit 54 to (english language and humans and yr="2015 - 2025") | 333 |

Database(s): **Embase**1974 to 2024 Week 46
Search Strategy:

| **#** | **Searches** | **Results** |
| --- | --- | --- |
| 1 | exp astrocytoma/ or exp diffuse intrinsic pontine glioma/ or exp ependymoma/ or exp gliosarcoma/ or exp oligodendroglioma/ | 132809 |
| 2 | Glioma*.tw. | 106658 |
| 3 | "isocitrate dehydrogenase mutation*" | 135 |
| 4 | "isocitrate dehydrogenase 1 mutation*" | 120 |
| 5 | "isocitrate dehydrogenase 2 mutation*" | 5 |
| 6 | "chromosome 1p and 19q codeletion*" | 1 |
| 7 | "1p/19q codeletion" | 367 |
| 8 | Astrocytoma*.tw. | 26018 |
| 9 | Glioblastoma*.tw. | 81366 |
| 10 | Gliosarcoma*.tw. | 1646 |
| 11 | ependymoma*.tw. | 9577 |
| 12 | oligodendroglioma*.tw. | 7345 |
| 13 | astroblastoma*.tw. | 338 |
| 14 | Xanthoastrocytoma*.tw. | 1120 |
| 15 | "Glial cell tumor*".tw. | 63 |
| 16 | "Glial tumor*".tw. | 2956 |
| 17 | exp Artificial Intelligence/ | 119916 |
| 18 | "Artificial Intelligence".tw. | 60183 |
| 19 | "Machine Learning".tw. | 132466 |
| 20 | "Deep Learning".tw. | 73371 |
| 21 | "Generative Pre-trained Transformer".tw. | 449 |
| 22 | "Generative Pre trained Transformer*".tw. | 488 |
| 23 | "Generative AI".tw. | 667 |
| 24 | "Large language model*".tw. | 3270 |
| 25 | "pre-trained transformer".tw. | 515 |
| 26 | "Decision Tree*".tw. | 24187 |
| 27 | "K-Nearest Neighbor*".tw. | 7353 |
| 28 | "Support vector machine*".tw. | 35134 |
| 29 | "Recurrent Neural Network*".tw. | 5468 |
| 30 | "Convolutional Neural Network*".tw. | 34499 |
| 31 | "Artificial neural network*".tw. | 22363 |
| 32 | "Deep Neural Network*".tw. | 12647 |
| 33 | "Naïve Bayes".tw. | 25 |
| 34 | "Naive Bayes".tw. | 4719 |
| 35 | "Bayesian Networks".tw. | 1891 |
| 36 | "Fuzzy Logic".tw. | 3136 |
| 37 | "K-Means".tw. | 11123 |
| 38 | "Random Forest*".tw. | 36208 |
| 39 | "Long Short-Term Memory".tw. | 5889 |
| 40 | "Gradient Boost*".tw. | 9890 |
| 41 | AdaBoost.tw. | 2071 |
| 42 | "Multilayer Perceptron".tw. | 3590 |
| 43 | "Ensemble learning".tw. | 2626 |
| 44 | "Generative Adversarial Network*".tw. | 4446 |
| 45 | "Transfer Learning".tw. | 6921 |
| 46 | histopatholog*.tw. | 420314 |
| 47 | patholog*.tw. | 1492928 |
| 48 | histolog*.tw. | 948856 |
| 49 | "whole-slide image*".tw. | 3562 |
| 50 | Immunohistochemistry.tw. | 344790 |
| 51 | 1 or 2 or 3 or 4 or 5 or 6 or 7 or 8 or 9 or 10 or 11 or 12 or 13 or 14 or 15 or 16 | 207375 |
| 52 | 17 or 18 or 19 or 20 or 21 or 22 or 23 or 24 or 25 or 26 or 27 or 28 or 29 or 30 or 31 or 32 or 33 or 34 or 35 or 36 or 37 or 38 or 39 or 40 or 41 or 42 or 43 or 44 or 45 | 395527 |
| 53 | 46 or 47 or 48 or 49 or 50 | 2798624 |
| 54 | 51 and 52 and 53 | 944 |
| 55 | limit 54 to (english language and humans and yr="2015 - 2025") | 828 |
| 56 | limit 55 to "remove medline records" | 462 |

| Database | Search Query | Hits |
| --- | --- | --- |
| CINAHL | AB (Glioma* OR "isocitrate dehydrogenase mutation*" OR "isocitrate dehydrogenase 1 mutation*" OR "isocitrate dehydrogenase 2 mutation*" OR "chromosome 1p and 19q codeletion*" OR "1p/19q codeletion" OR Astrocytoma* OR Glioblastoma* OR Gliosarcoma* OR ependymoma* OR oligodendroglioma* OR astroblastoma* OR Xanthoastrocytoma* OR "glial tumor*" OR "Glial cell tumor*" ) AND AB ( "Artificial Intelligence" OR "Machine Learning" OR "Deep Learning" OR "Generative Pre-trained Transformer" OR "Generative Pre trained Transformer*" OR "Generative AI" OR "Large language model*" OR "pre-trained transformer" OR "Decision Tree*" OR "K-Nearest Neighbor*" OR "Support vector machine*" OR "Recurrent Neural Network*" OR "Convolutional Neural Network*" OR "Artificial neural network*" OR "Deep Neural Network*" OR "Naïve Bayes" OR "Naive Bayes" OR "Bayesian Networks" OR "Fuzzy Logic" OR "K-Means" OR "Random Forest*" OR "Long Short-Term Memory" OR "Gradient Boost*" OR AdaBoost OR "Multilayer Perceptron" OR "Ensemble learning" OR "Generative Adversarial Network*" OR "Transfer Learning" ) AND AB ( histopatholog* OR patholog* OR histolog* OR "whole-slide image*" OR Immunohistochemistry ) Limiters - Date Published: 20150101-20241231 Expanders - Apply equivalent subjects Narrow by Language: - english | 76 |
| Scopus | ( TITLE-ABS-KEY ( glioma* OR "isocitrate dehydrogenase mutation*" OR "isocitrate dehydrogenase 1 mutation*" OR "isocitrate dehydrogenase 2 mutation*" OR "chromosome 1p and 19q codeletion*" OR "1p/19q codeletion" OR astrocytoma* OR glioblastoma* OR gliosarcoma* OR ependymoma* OR oligodendroglioma* OR astroblastoma* OR xanthoastrocytoma* OR "glial tumor*" OR "Glial cell tumor*" ) AND TITLE-ABS-KEY ( "Artificial Intelligence" OR "Machine Learning" OR "Deep Learning" OR "Generative Pre-trained Transformer" OR "Generative Pre trained Transformer*" OR "Generative AI" OR "Large language model*" OR "pre-trained transformer" OR "Decision Tree*" OR "K-Nearest Neighbor*" OR "Support vector machine*" OR "Recurrent Neural Network*" OR "Convolutional Neural Network*" OR "Artificial neural network*" OR "Deep Neural Network*" OR "Naïve Bayes" OR "Naive Bayes" OR "Bayesian Networks" OR "Fuzzy Logic" OR "K-Means" OR "Random Forest*" OR "Long Short-Term Memory" OR "Gradient Boost*" OR adaboost OR "Multilayer Perceptron" OR "Ensemble learning" OR "Generative Adversarial Network*" OR "Transfer Learning" ) AND TITLE-ABS-KEY ( histopatholog* OR patholog* OR histolog* OR "whole-slide image*" OR immunohistochemistry ) ) AND PUBYEAR > 2014 AND PUBYEAR < 2026 AND ( LIMIT-TO ( DOCTYPE , "ar" ) OR LIMIT-TO ( DOCTYPE , "cp" ) ) AND ( EXCLUDE ( EXACTKEYWORD , "Animals" ) OR EXCLUDE ( EXACTKEYWORD , "Animal" ) ) AND ( LIMIT-TO ( LANGUAGE , "English" ) ) | 1430 |
| ACM Digital Library | [[Abstract: glioma*] OR [Abstract:"isocitrate dehydrogenase mutation*"] OR [Abstract:"isocitrate dehydrogenase 1 mutation*"] OR [Abstract:"isocitrate dehydrogenase 2 mutation*"] OR [Abstract:"chromosome 1p and 19q codeletion*"] OR [Abstract:"1p/19q codeletion"] OR [Abstract: astrocytoma*] OR [Abstract: glioblastoma*] OR [Abstract: gliosarcoma*] OR [Abstract: ependymoma*] OR [Abstract: oligodendroglioma*] OR [Abstract: astroblastoma*] OR [Abstract: xanthoastrocytoma*] OR [Abstract: "glial tumor*"] OR [Abstract: "glial cell tumor*"]] AND [[Abstract: "artificial intelligence"] OR [Abstract: "machine learning"] OR [Abstract: "deep learning"] OR [Abstract: "generative pre-trained transformer"] OR [Abstract: "generative pre trained transformer*"] OR [Abstract: "generative ai"] OR [Abstract: "large language model*"] OR [Abstract: "pre-trained transformer"] OR [Abstract: "decision tree*"] OR [Abstract: "k-nearest neighbor*"] OR [Abstract: "support vector machine*"] OR [Abstract: "recurrent neural network*"] OR [Abstract: "convolutional neural network*"] OR [Abstract: "artificial neural network*"] OR [Abstract: "deep neural network*"] OR [Abstract: "naïve bayes"] OR [Abstract: "naive bayes"] OR [Abstract: "bayesian networks"] OR [Abstract: "fuzzy logic"] OR [Abstract: "k-means"] OR [Abstract: "random forest*"] OR [Abstract: "long short-term memory"] OR [Abstract: "gradient boost*"] OR [Abstract: adaboost] OR [Abstract: "multilayer perceptron"] OR [Abstract: "ensemble learning"] OR [Abstract: "generative adversarial network*"] OR [Abstract: "transfer learning"]] AND [[All: histopatholog*] OR [All: patholog*] OR [All: histolog*] OR [All: "whole-slide image*"] OR [All: immunohistochemistry]] | 11 |
| IEEE Xplore | ("Abstract":Glioma* OR "Abstract":"isocitrate dehydrogenase mutation*" OR "Abstract":"isocitrate dehydrogenase 1 mutation*" OR "Abstract":"isocitrate dehydrogenase 2 mutation*" OR "Abstract":"chromosome 1p and 19q codeletion*" OR "Abstract":"1p/19q codeletion" OR "Abstract":Astrocytoma* OR "Abstract":Glioblastoma* OR "Abstract":Gliosarcoma* OR "Abstract":ependymoma* OR "Abstract":oligodendroglioma* OR "Abstract":astroblastoma* OR "Abstract":Xanthoastrocytoma* OR "Abstract":"glial tumor*" OR "Abstract":"Glial cell tumor*") AND ("Abstract":"Artificial Intelligence" OR "Abstract":"Machine Learning" OR "Abstract":"Deep Learning" OR "Abstract":"Generative Pre-trained Transformer" OR "Abstract":"Generative Pre trained Transformer" OR "Abstract":"Generative Pre trained Transformers" OR "Abstract":"Generative AI" OR "Abstract":"Large language model" OR "Abstract":"Large language models" OR "Abstract":"pre-trained transformer" OR "Abstract":"Decision Tree" OR "Abstract":"K-Nearest Neighbor" OR "Abstract":"Support vector machine" OR "Abstract":"Recurrent Neural Network" OR "Abstract":"Recurrent Neural Networks" OR "Abstract":"Convolutional Neural Network" OR "Abstract":"Convolutional Neural Networks" OR "Abstract":"Artificial neural network" OR "Abstract":"Artificial neural networks" OR "Abstract":"Deep Neural Network" OR "Abstract":"Deep Neural Networks" OR "Abstract":"Naïve Bayes" OR "Abstract":"Naive Bayes" OR "Abstract":"Bayesian Networks" OR "Abstract":"Fuzzy Logic" OR "Abstract":"K-Means" OR "Abstract":"Random Forest" OR "Abstract":"Long Short-Term Memory" OR "Abstract":"Gradient Boost" OR "Abstract":AdaBoost OR "Abstract":"Multilayer Perceptron" OR "Abstract":"Ensemble learning" OR "Abstract":"Generative Adversarial Network" OR "Abstract":"Generative Adversarial Networks" OR "Abstract":"Transfer Learning") AND ("Abstract":Histopathology OR "Abstract":Histopathological OR "Abstract":pathology OR "Abstract":pathological OR "Abstract":histology OR "Abstract":histological OR "Abstract":"whole-slide image" OR "Abstract":"whole-slide images" OR "Abstract":Immunohistochemistry)  Filters Applied: 2015 - 2024 | 41 |
| Google Scholar | (Glioma* OR "isocitrate dehydrogenase mutation*" OR "isocitrate dehydrogenase 1 mutation*" OR "isocitrate dehydrogenase 2 mutation*" OR "chromosome 1p and 19q codeletion*" OR "1p/19q codeletion" OR Astrocytoma* OR Glioblastoma* OR Gliosarcoma* OR ependymoma* OR oligodendroglioma* OR astroblastoma* OR Xanthoastrocytoma* OR "glial tumor*" OR "Glial cell tumor*") AND ("Artificial Intelligence" OR "Machine Learning" OR "Deep Learning" OR "Generative Pre-trained Transformer" OR "Generative Pre trained Transformer*" OR "Generative AI" OR "Large language model*" OR "pre-trained transformer" OR "Decision Tree*" OR "K-Nearest Neighbor*" OR "Support vector machine*" OR "Recurrent Neural Network*" OR "Convolutional Neural Network*" OR "Artificial neural network*" OR "Deep Neural Network*" OR "Naïve Bayes" OR "Naive Bayes" OR "Bayesian Networks" OR "Fuzzy Logic" OR "K-Means" OR "Random Forest*" OR "Long Short-Term Memory" OR "Gradient Boost*" OR AdaBoost OR "Multilayer Perceptron" OR "Ensemble learning" OR "Generative Adversarial Network*" OR "Transfer Learning") AND (histopatholog* OR patholog* OR histolog* OR "whole-slide image*" OR Immunohistochemistry) | 100 |
