# Supplementary material for: The Performance of Artificial Intelligence in Classifying Molecular Markers in Adult-Type Gliomas Using Histopathological Images: Systematic Review
Source: J Med Internet Res. 2026 Mar 13;28:e78377. doi: 10.2196/78377 (PMC12986776; doi:10.2196/78377)
Supplement: Multimedia Appendix 2 [file jmir-v28-e78377-s002.docx]

| **Extracted data** | **Definition** |
| --- | --- |
| **Study Characteristics** |  |
| Author | The first author of the study (Surname name). |
| Year of publication | The year in which the study was published. |
| Country of publication | First author affiliation. |
| Type of publication | The venue where the study was published: peer-reviewed journal articles, conference papers, or dissertations. |
| **Participants and Dataset Characteristics** |  |
| Number of participants | What was the number of participants from which the data was collected? |
| Mean Age | What is the mean age of the participants? |
| Female percentage | What is the female percentage of the participants? |
| Dataset size | What was the total number of histopathological images collected from participants to develop the model? |
| Data sources | What was the source of data that was used for developing the algorithms (open source or closed source)? |
| Open Dataset name | What was the name of open data source that was used for developing the algorithms (TCGA, DBTA, ..)? |
| Magnification of images | What magnification level was used for the histopathological images in the study (e.g., 40x, 100x, etc..)? |
| Slide images resolution | What is the slide resolution used (80,000_80,000 pixels, .. )? |
| Level of image analysis | What was the level of image analysis in the study: architecture level, cell level, or both? (Architectural level (1.25x–20x), Cellular level (20x–40x and above)) |
| Number of classes | For classification problems, how many classes were available in the dataset? |
| **AI** **Characteristics** |  |
| AI classifiers | What are the AI classifiers (e.g., RF, SVM, ANN, CNN, RNN, DNN, k-NN, MLP, DBN, DBM, DPN BN, CRT, DT, LASSO, LR, MFA, MLR, MDL, NB, NN, NSC, RBFN) used in the paper? |
| Aim of classifier | What was the algorithm used for (Detection or Subtyping)? Detection of IDH mutations or Subtyping of the IDH mutation status to mutant and wildtype? Subtyping of the 1p/19q gene deletion (co-deleted, non-co-deleted)? |
| Specific classifier | What was the Specific algorithm used for (Detection or Subtyping)? |
| Type of classification | What type of classification was used in the study: binary or multiclass classification? (ResNet50, VGG19, InceptionV3, InceptionResNetV2, Logistic regression, Traditional MIL, MLP, ..) |
| Data input to AI algorithm | What data was used for developing the AI algorithm: histopathological images, clinical data, and/or others? |
| Features extraction methods | What method was employed to extract features from histopathological images (CNN, Textural feature analysis, Color feature analysis, Morphological feature analysis, auto-encoding unsupervised feature learning (UFL))? |
| Specific features extraction model | What Specific method was employed to extract features from histopathological images (ResNet50, VGG19, InceptionV3, InceptionResNetV2, Logistic regression, Traditional MIL, MLP, ..) |
| Type of validation | What validation approach was used for the developed algorithm (e.g., training-test split, K-fold cross-validation, nested cross-validation, leave-one-out cross-validation, apparent validation, external validation)? |
| Performance metrics | What performance metrics were assessed in the study (e.g., accuracy, sensitivity, specificity, etc.)? |
| **Results of studies** |  |
| Number of cases | What is the number of images that showed no IDH mutation or mutant type? and wildtype? |
| Number of controls | What is the number of images that showed the presence of IDH mutation or wildtype? |
| Confusion Matrix | What are the confusion matrix estimates for the highest estimate for each algorithm: true positive, true negative, false positive, false negative |
| Highest accuracy | What is the highest accuracy for each algorithm? |
| Highest sensitivity | What is the highest sensitivity for each algorithm? |
| Highest specificity | What is the highest specificity for each algorithm? |
| Highest AUC | What is the highest AUC for each algorithm? |
