# Supplementary material for: The Performance of Artificial Intelligence in Classifying Molecular Markers in Adult-Type Gliomas Using Histopathological Images: Systematic Review
Source: J Med Internet Res. 2026 Mar 13;28:e78377. doi: 10.2196/78377 (PMC12986776; doi:10.2196/78377)
Supplement: Multimedia Appendix 3 [file jmir-v28-e78377-s003.docx]

Multimedia Appendix 4: The modifed version of QUADAS-2

| **1. Participants** | **Signaling questions** | **Explanation** |
| --- | --- | --- |
|  | 1.1 Was a consecutive or random sample of patients enrolled? | -**Yes**: if a consecutive or random sample of eligible patients was enrolled.  - **No**: if patients were selected by convenience;  - **Unclear**: if the study did not report the manner in which participants were enrolled or reproted unclear selection criteria. |
|  | 1.2 Did the study avoid inappropriate exclusions? | - **Yes**: If inclusion and exclusion of participants were appropriate, ensuring a representative sample.  - **No**: If participants were excluded in a way that could bias model performance (e.g., excluding high-risk groups).  - **Unclear**: When there is no information on whether inappropriate inclusions or exclusions took place. |
|  | 1.3 Was the sample size sufficient? | - **Yes**: If at least 100 participants were included, or if studies with multiple samples per participant had at least 100 samples.  - **No**: If fewer than 100 participants or samples were included.  - **Unclear**: If the sample size was not clearly reported. |
|  | 1.4 Was there a balance in the number of patients between the subgroups? | - **Yes**: if the percentage of participants in any group is 66.7% or less of the total sample (≤2/3).  - **No**: if the percentage of participants in any group is more than 66.7% of the total sample (>2/3).  - **Unclear**: If no information was provided regarding the number of participants in the groups. |
|  | **Risk-of-bias assessment:** Could the selection of participants have introduced bias? | - **Low risk of bias**: If the answer to all signaling questions is ‘Yes’.  - **High risk of bias**: If the answer to any of the signaling questions is “No”.  - **Unclear risk of bias**: If the answer to all signaling questions is “Unclear”.  Or if the answer to some signaling questions is “Unclear” and none of the answers to signaling questions is “No”. |
|  | **Concerns regarding applicability:** Are there concerns that the included participants and setting do not match the review question? | - **Low concern for applicability**: If the study population aligns with the target population of the review.  - **High concern for applicability**: If the study includes a non-representative population.  - **Unclear concern for applicability**: If there is insufficient information available to make a judgment about the applicability |
| **2. Index test (AI models)** | 2.1 Were the AI models described in detail? | -**Yes**: if the model details were provided (e.g., model type, architecture, hyperparameters, features used, number of layers, pooling, normalization, regularization, and activation in the layers, etc.)  or if a previously published model is  employed, the paper must cite a reference that meets the preceding standards and fully describe every modification made to the model.  - **No**: if only the model’s name was reported in the paper, or the study reported some information but other important information still missing.  - **Unclear**: If the study reported some details but they are not clear. |
|  | 2.2 Were all features (predictors) used in the model clearly identified? | - **Yes**: If all features used in each model were clearly reported.  - **No**: If any features used in any model were not reported.  - **Unclear**: If details provided about the features are not clear. |
|  | 2.3 Were features assessed in the same way for all participants? | - **Yes**: If the assessment of features were similar for all participants (all participants underwent standardized data collection).  - **No**: If different methods/devices were used without standardization (e.g., multiple types of hitopathological images).  - **Unclear**: If assessment methods were not reported. |
|  | 2.4 Were features collected without knowledge of outcome? | - **Yes**: If features (e.g., histopathological images) were collected without knowledge of outcome (diagnosis/label).  - **No**: if features (e.g., histopathological images) were collected with knowledge of outcome (diagnosis/label).  - **Unclear**: No information on whether features were assessed without knowledge of outcome information. |
|  | **Risk-of-bias assessment:** Could the conduct or interpretation of the index test have introduced bias? | - **Low risk of bias**: If the answer to all signaling questions is ‘Yes’.  - **High risk of bias**: If the answer to any of the signaling questions is “No”.  - **Unclear risk of bias**: If the answer to all signaling questions is “Unclear”.  Or if the answer to some signaling questions is “Unclear” and none of the answers to signaling questions is “No”. |
|  | **Concerns regarding applicability:** Are there concerns that the definition, assessment, or timing of the index test in the model does not match the review question? | - **Low concern for applicability**: Definition, assessment, and timing of predictors match the review question.  - **High concern for applicability**: Definition, assessment, or timing of predictors were different from the review question.  - **Unclear concern for applicability**: If relevant information about the predictors is not reported. |
| **3. Reference Standard (Ground truth)** | 3.1 Was the reference standard likely to correctly classify the outcome? | Were the assessors/annotators qualified?  **- Yes:** If histopathology images were analyzed by an expert (e.g., pathologist or neuropathologist), ensuring that the individual has the necessary expertise and experience to accurately classify the outcome.  **- No:** If histopathology images were analyzed by individuals without specific expertise in pathology (e.g., general physicians, non-specialist technicians, or automated systems without validation).  **- Unclear:** If information about the assessors or annotators are not specified. |
|  | 3.2 Was the outcome defined and determined in a similar way for all participants? | - **Yes**: If outcomes were defined and determined in a similar way for all participants (i.e., the same diagnostic criteria were applied to all participants)  - **No**: If outcomes were clearly defined and determined in a different way for some participants (i.e., different criteria were applied).  - **Unclear**: No information on whether outcomes were defined or determined in a similar way for all participants. |
|  | 3.3 Was the outcome determined without knowledge of predictor data? | - **Yes**: If outcome classification (i.e., images labelling) was blinded to AI model predictions.  - **No**: If assessors had access to AI-generated predictions when classifying outcomes.  - **Unclear**: No information on whether the outcome was determined without knowledge of predictor data. |
|  | **Risk-of-bias assessment:** Could the reference standard, its conduct, or its interpretation have introduced bias? | - **Low risk of bias**: If the answer to all signaling questions is ‘Yes’.  - **High risk of bias**: If the answer to any of the signaling questions is “No”.  - **Unclear risk of bias**: If the answer to all signaling questions is “Unclear”.  Or if the answer to some signaling questions is “Unclear” and none of the answers to signaling questions is “No”. |
|  | **Concerns regarding applicability:** Are there concerns that the outcome definition, timing, or determination do not  match the review question? | - **Low concern for applicability**: Outcome definition, timing, and method of determination defines the outcome as intended by the review question.  -**High concern for applicability**: Choice of outcome definition, timing, and method of outcome determination defines another outcome as intended by the review question.  - **Unclear concern for applicability**: If relevant information about the outcome, timing, and method of determination is not reported. |
| **4.** **Analysis** | 4.1 Were all participants included in the analysis? | - **Yes**: If all participants enrolled in the study are  included in the data analysis.  - **No**: If some or a subgroup of participants are  inappropriately excluded without justification.  - **Unclear**: No information on whether all enrolled participants are included in the analysis. |
|  | 4.2 Was data preprocessing carried out appropriately? | - **Yes**: If there were no missing values, or if missing values were handled appropriately (e.g., multiple imputation).  - **No**: If participants with missing data are omitted, or if the method of handling missing data is clearly flawed (e.g., missing indicator method or inappropriate use of last value carried forward).  - **Unclear**: If there is insufficient information to determine if the method of handling missing data is appropriate. |
|  | 4.3 Was the breakdown of the training, validation, and test sets appropriate? | - **Yes**: If there's a clear rationale for the chosen distribution and it aligns with best practices in the field (e.g., 70-80% training, 10-15% validation, 10-20% test).  - **No**: if the division did not aligns with best practices in the field without a valid reason.  - **Unclear**: If data splitting details were missing. |
|  | 4.4 Was the performance of the model evaluated appropriately? | - **Yes**: If the confusion matrix was presented,  Or more than one measure was used and the selected measures were appropriate.  - **No**: If the confusion matrix was not presented, and only one measure was reported,  Or the selected measures were not appropriate.  - **Unclear**: If no information was provided on the performance measures |
|  | **Risk-of-bias assessment:** Could the analysis, its conduct, or its interpretation have introduced bias? | - **Low risk of bias**: If the answer to all signaling questions is ‘Yes’.  - **High risk of bias**: If the answer to any of the signaling questions is “No”.  - **Unclear risk of bias**: If the answer to all signaling questions is “Unclear”.  Or if the answer to some signaling questions is “Unclear” and none of the answers to signaling questions is “No”. |
