# Supplementary material for: The Performance of Artificial Intelligence in Classifying Molecular Markers in Adult-Type Gliomas Using Histopathological Images: Systematic Review
Source: J Med Internet Res. 2026 Mar 13;28:e78377. doi: 10.2196/78377 (PMC12986776; doi:10.2196/78377)
Supplement: Multimedia Appendix 5 [file jmir-v28-e78377-s005.docx]

Multimedia Appendix 6: Features of histopathological images in each study

| Study ^Ref^ | Data source | Open Dataset name | Magnification | Slide image resolution | Level of analysis | Number of classes deletion |
| --- | --- | --- | --- | --- | --- | --- |
| Albuquerque^1^ | Open source | TCGA | NR | 512 × 512 px, 2048 × 2048 px | NR | 3 |
| Chitnis^2^ | Open source | DBTA | 20x | 256×256 patches | Architectural level, Cellular level | 2 |
| Cui^3^ | Open source | TCGA | NR | 32x32 px, 64x64 px, 128x128 px, 256x256 px, 512x512 px, 1024×1024 px | NR | 2 |
| Despotovic^4^ | Closed source | NA | 10x, 20x, 40x, 100x | 100000x100000  px | Architectural level, Cellular level | 5 |
| Fang^5^ | Open source, Closed source | TCGA | 20x | 256×256 patches | Architectural level, Cellular level | 4 |
| Faust^6^ | Closed source | NA | 20x | 100 × 100 px | Architectural level, Cellular level | 3 |
| Hewitt^7^ | Open source, Closed source | TCGA | NR | NR | NR | 4 |
| Innani^8^ | Open source | TCGA | 20x | 256 x 256 patches | Architectural level, Cellular level | 2 |
| Jiang^9^ | Open source | TCGA | 10x | 224x224 px | Architectural level | 2 |
| Jungo^10^ | Closed source | NA | 40x | 1920 x 1017 px | Cellular level | 2 |
| Kim^11^ | Open source, Closed source | TCGA | 20x, 40x | 224 × 224 | Architectural level, Cellular level | 2 |
| Krebs^12^ | Open source | TCGA | 5x, 20x | 256 x 256 px | Architectural level, Cellular level | 2 |
| Li^13^ | Open source, Closed source | TCGA | NR | 1024x1024 megapixel patches | NR | 2, 5 |
| Liechty^14^ | Open source, Closed source | TCGA | 2.5×, 5×, 10×, 20× | 256×256 px patches | Architectural level, Cellular level | 2 |
| Liu^15^ | Closed source | NA | 20x, 40x | 1024x1024 patches | Architectural level, Cellular level | 2 |
| Liu^16^ | Open source, Closed source | TCGA | 10x | 256×256 px, 1024×1024 px | Architectural level | 2 |
| Nakagaki^17^ | Open source | TCGA | NR | 256×256 px, 224 × 224 px (for CoAtNet) | NR | 2 |
| Pei^18^ | Open source | TCGA | NR | 512x512 patches | NR | 4 |
| Rathore^19^ | Open source | TCGA | NR | NR | NR | 4 |
| Wang^20^ | Closed source | NA | 20x | 1024×1024 px | Architectural level, Cellular level | 6 |
| Wang^21^ | Open source, Closed source | TCGA | 20x, 40x | 5120x5120 | Architectural level, Cellular level | 2 |
| Zhao^22^ | Closed source | NA | 20x | 2048×2048 patches, 224x224 patches resized | Architectural level, Cellular level | 2 |
| TCGA: The Cancer Genome Atlas, DBTA: Digital Brain Tumor Atlas, NR: Not reported | | | | | | |

**References** :

1. Ostrom, Q.T., Cioffi, G., Waite, K., Kruchko, C., Barnholtz-Sloan, J.S.: CBTRUS Statistical Report: Primary Brain and Other Central Nervous System Tumors Diagnosed in the United States in 2014–2018. Neuro Oncol. 23, iii1 (2021). doi: 10.1093/neuonc/noab200

2. Reynoso-Noverón, N., Mohar-Betancourt, A., Ortiz-Rafael, J.: Epidemiology of brain tumors. Principles of Neuro-Oncology: Brain & Skull Base. 15–25 (2020). doi: 10.1007/978-3-030-54879-7_2

3. Lapointe, S., Perry, A., Butowski, N.A.: Primary brain tumours in adults. Lancet. 392, 432–446 (2018). doi: 10.1016/S0140-6736(18)30990-5

4. Perry, A., Wesseling, P.: Histologic classification of gliomas. Handb Clin Neurol. 134, 71–95 (2016). doi: 10.1016/B978-0-12-802997-8.00005-0

5. Chen, R., Smith-Cohn, M., Cohen, A.L., Colman, H.: Glioma Subclassifications and Their Clinical Significance. Neurotherapeutics. 14, 284 (2017). doi: 10.1007/s13311-017-0519-x

6. N, K., R, O., S, B., W, M.: Molecular Classification of Diffuse Gliomas. Can J Neurol Sci. 47, 464–473 (2020). doi: 10.1017/cjn.2020.107. Antonelli, M., Poliani, P.L.: Adult type diffuse gliomas in the new 2021 WHO Classification. Pathologica. 114, 397 (2022). doi: 10.32074/1591-951x-8238. Hollon, T., Jiang, C., Chowdury, A., Nasir-Moin, M., Kondepudi, A., Aabedi, A., Adapa, A., Al-Holou, W., Heth, J., Sagher, O., Lowenstein, P., Castro, M., Wadiura, L.I., Widhalm, G., Neuschmelting, V., Reinecke, D., von Spreckelsen, N., Berger, M.S., Hervey-Jumper, S.L., Golfinos, J.G., Snuderl, M., Camelo-Piragua, S., Freudiger, C., Lee, H., Orringer, D.A.: Artificial-intelligence-based molecular classification of diffuse gliomas using rapid, label-free optical imaging. Nat Med. 29, 828–832 (2023). doi: 10.1038/s41591-023-02252-4

9. Koski, E., Murphy, J.: AI in Healthcare. Stud Health Technol Inform. 284, 295–299 (2021). doi: 10.3233/shti210726

10. van Kempen, E.J., Post, M., Mannil, M., Kusters, B., Ter Laan, M., Meijer, F.J.A., Henssen, D.J.H.A.: Accuracy of machine learning algorithms for the classification of molecular features of gliomas on mri: A systematic literature review and meta-analysis. Cancers (Basel). 13, 2606 (2021). doi: 10.3390/cancers13112606

11. Farahani, S., Hejazi, M., Moradizeyveh, S., Di Ieva, A., Fatemizadeh, E., Liu, S.: Diagnostic Accuracy of Deep Learning Models in Predicting Glioma Molecular Markers: A Systematic Review and Meta-Analysis. Diagnostics. 15, 797 (2025). doi: 10.3390/diagnostics15070797

12. Puustinen, S., Vrzáková, H., Hyttinen, J., Rauramaa, T., Fält, P., Hauta-Kasari, M., Bednarik, R., Koivisto, T., Rantala, S., von und zu Fraunberg, M., Jääskeläinen, J.E., Elomaa, A.P.: Hyperspectral Imaging in Brain Tumor Surgery—Evidence of Machine Learning-Based Performance. World Neurosurg. 175, e614–e635 (2023). doi: 10.1093/nop/npad040

13. Singh, J., Sahu, S., Mohan, T., Mahajan, S., Sharma, M.C., Sarkar, C., Suri, V.: Current status of DNA methylation profiling in neuro-oncology as a diagnostic support tool: A review. Neurooncol Pract. 10, 518–526 (2023). doi: 10.1093/nop/npad040

14. Liu, Y., Wu, M.: Deep learning in precision medicine and focus on glioma. Bioeng Transl Med. 8, e10553 (2023). doi: 10.1002/btm2.1055315. Zhao, J., Huang, Y., Song, Y., Xie, D., Hu, M., Qiu, H., Chu, J.: Diagnostic accuracy and potential covariates for machine learning to identify IDH mutations in glioma patients: evidence from a meta-analysis. Eur Radiol. 30, 4664–4674 (2020). doi: 10.1007/s00330-020-06717-9

16. Lv, Q., Liu, Y., Sun, Y., Wu, M.: Insight into deep learning for glioma IDH medical image analysis: A systematic review. Medicine. 103, E37150 (2024). doi: 10.1097/md.0000000000037150

17. Guha, A., Halder, S., Shinde, S.H., Gawde, J., Munnolli, S., Talole, S., Goda, J.S.: How does deep learning/machine learning perform in comparison to radiologists in distinguishing glioblastomas (or grade IV astrocytomas) from primary CNS lymphomas?: a meta-analysis and systematic review. Clin Radiol. 79, 460–472 (2024). doi: 10.1016/j.crad.2024.03.007

18. McInnes, M.D.F., Moher, D., Thombs, B.D., McGrath, T.A., Bossuyt, P.M., Clifford, T., Cohen, J.F., Deeks, J.J., Gatsonis, C., Hooft, L., Hunt, H.A., Hyde, C.J., Korevaar, D.A., Leeflang, M.M.G., Macaskill, P., Reitsma, J.B., Rodin, R., Rutjes, A.W.S., Salameh, J.P., Stevens, A., Takwoingi, Y., Tonelli, M., Weeks, L., Whiting, P., Willis, B.H.: Preferred Reporting Items for a Systematic Review and Meta-analysis of Diagnostic Test Accuracy Studies: The PRISMA-DTA Statement. JAMA. 319, 388–396 (2018). doi: 10.1001/jama.2017.19163

19. Whiting, P.F., Rutjes, A.W.S., Westwood, M.E., Mallett, S., Deeks, J.J., Reitsma, J.B., Leeflang, M.M.G., Sterne, J.A.C., Bossuyt, P.M.M.: Quadas-2: A revised tool for the quality assessment of diagnostic accuracy studies. Ann Intern Med. 155, 529–536 (2011). doi: 10.7326/0003-4819-155-8-201110180-00009

20. Wolff, R.F., Moons, K.G.M., Riley, R.D., Whiting, P.F., Westwood, M., Collins, G.S., Reitsma, J.B., Kleijnen, J., Mallett, S.: PROBAST: A tool to assess the risk of bias and applicability of prediction model studies. Ann Intern Med. 170, 51–58 (2019). doi: 10.7326/m18-1376

21. Albuquerque, T., Fang, M.L., Wiestler, B., Delbridge, C., Vasconcelos, M.J.M., Cardoso, J.S., Schüffler, P.: Multimodal Context-Aware Detection of Glioma Biomarkers Using MRI and WSI. In: Medical Image Computing and Computer Assisted Intervention – MICCAI 2023 Workshops. pp. 157–167 (2023) https://doi.org/10.1007/978-3-031-47425-5_15

22. Chitnis, S.R., Liu, S., Dash, T., Verlekar, T.T., Di Ieva, A., Berkovsky, S., Vig, L., Srinivasan, A.: Domain-Specific Pre-training Improves Confidence in Whole Slide Image Classification. Proceedings of the Annual International Conference of the IEEE Engineering in Medicine and Biology Society, EMBS. (2023). doi: 10.1109/embc40787.2023.10340659

23. Cui, D., Liu, Y., Liu, G., Liu, L.: A Multiple-Instance Learning-Based Convolutional Neural Network Model to Detect the IDH1 Mutation in the Histopathology Images of Glioma Tissues. J Comput Biol. 27, 1264–1272 (2020). doi: 10.1089/cmb.2019.041024. Despotovic, V., Kim, S.Y., Hau, A.C., Kakoichankava, A., Klamminger, G.G., Borgmann, F.B.K., Frauenknecht, K.B.M., Mittelbronn, M., Nazarov, P. V: Glioma subtype classification from histopathological images using in-domain and out-of-domain transfer learning: An experimental study. Heliyon. 10, e27515 (2024). doi: 10.1016/j.heliyon.2024.e27515

25. Fang, Z., Liu, Y., Wang, Y., Zhang, X., Chen, Y., Cai, C., Lin, Y., Han, Y., Wang, Z., Zeng, S., Shen, H., Tan, J., Zhang, Y.: Deep Learning Predicts Biomarker Status and Discovers Related Histomorphology Characteristics for Low-Grade Glioma. (2023) https://doi.org/10.48550/arXiv.2310.07464

26. Faust, K., Lee, M.K., Dent, A., Fiala, C., Portante, A., Rabindranath, M., Alsafwani, N., Gao, A., Djuric, U., Diamandis, P.: Integrating morphologic and molecular histopathological features through whole slide image registration and deep learning. Neurooncol Adv. 4, vdac001 (2022). doi: 10.1093/noajnl/vdac001

27. Hewitt, K.J., Loffler, C.M.L., Muti, H.S., Berghoff, A.S., Eisenloffel, C., van Treeck, M., Carrero, Z.I., El Nahhas, O.S.M., Veldhuizen, G.P., Weil, S., Saldanha, O.L., Bejan, L., Millner, T.O., Brandner, S., Bruckmann, S., Kather, J.N.: Direct image to subtype prediction for brain tumors using deep learning. Neurooncol Adv. 5, vdad139 (2023). doi: 10.1093/noajnl/vdad139

28. Innani, S., Baheti, B., Nasrallah, M.P., Bakas, S.: Weakly Supervised IDH-Status Glioma Classification from H&E-Stained Whole Slide Images, (2024) doi: 10.1109/ISBI56570.2024.10635869

29. Jiang, S., Zanazzi, G.J., Hassanpour, S.: Predicting prognosis and IDH mutation status for patients with lower-grade gliomas using whole slide images. Sci Rep. 11, 16849 (2021). doi: 10.1038/s41598-021-95948-x

30. Jungo, P., Hewer, E.: Code-free machine learning for classification of central nervous system histopathology images. J Neuropathol Exp Neurol. 82, 221–230 (2023). doi: 10.1093/jnen/nlac131

31. Kim, G.J., Lee, T., Ahn, S., Uh, Y., Kim, S.H.: Efficient diagnosis of IDH-mutant gliomas: 1p/19qNET assesses 1p/19q codeletion status using weakly-supervised learning. NPJ Precis Oncol. 7, 94 (2023). doi: 10.1038/s41698-023-00450-4

32. Krebs, O., Agarwal, S., Tiwari, P., Tomaszewski, J.E., Ward, A.D.: Self-supervised deep learning to predict molecular markers from routine histopathology slides for high-grade glioma tumors, (2023) doi:10.1117/12.2653929

33. Li, Z., Cong, Y., Chen, X., Qi, J., Sun, J., Yan, T., Yang, H., Liu, J., Lu, E., Wang, L., Li, J., Hu, H., Zhang, C., Yang, Q., Yao, J., Yao, P., Jiang, Q., Liu, W., Song, J., Carin, L., Chen, Y., Zhao, S., Gao, X.: Vision transformer-based weakly supervised histopathological image analysis of primary brain tumors. iScience. 26, 105872 (2023). doi: 10.1016/j.isci.2022.105872

34. Liechty, B., Xu, Z., Zhang, Z., Slocum, C., Bahadir, C.D., Sabuncu, M.R., Pisapia, D.J.: Machine learning can aid in prediction of IDH mutation from H&E-stained histology slides in infiltrating gliomas. Sci Rep. 12, 22623 (2022). doi: 10.1038/s41598-022-26170-635. Liu, X., Hu, W., Diao, S., Abera, D.E., Racoceanu, D., Qin, W.: Multi-scale feature fusion for prediction of IDH1 mutations in glioma histopathological images. Comput Methods Programs Biomed. 248, 108116 (2024). doi: 10.1016/j.cmpb.2024.108116

36. Liu, S., Shah, Z., Sav, A., Russo, C., Berkovsky, S., Qian, Y., Coiera, E., Di Ieva, A.: Isocitrate dehydrogenase (IDH) status prediction in histopathology images of gliomas using deep learning. Sci Rep. 10, 7733 (2020). doi: 10.1038/s41598-020-64588-y37. Nakagaki, R., Debsarkar, S.S., Kawanaka, H., Aronow, B.J., Prasath, V.B.S.: Deep learning-based IDH1 gene mutation prediction using histopathological imaging and clinical data. Comput Biol Med. 179, 108902 (2024). doi: 10.1016/j.compbiomed.2024.108902

38. Pei, L., Jones, K.A., Shboul, Z.A., Chen, J.Y., Iftekharuddin, K.M.: Deep Neural Network Analysis of Pathology Images With Integrated Molecular Data for Enhanced Glioma Classification and Grading. Front Oncol. 11, 668694 (2021). doi: 10.3389/fonc.2021.668694

39. Rathore, S., Iftikhar, M., Nasrallah, M., Gurcan, M., Rajpoot, N., Mourelatos, Z.: Prediction of overall survival and molecular markers in gliomas via analysis of digital pathology images using deep learning. Neuro Oncol. 21, vi270–vi270 (2019). doi: 10.1093/neuonc/noz175.1134

40. Wang, W., Zhao, Y., Teng, L., Yan, J., Guo, Y., Qiu, Y., Ji, Y., Yu, B., Pei, D., Duan, W., Wang, M., Wang, L., Duan, J., Sun, Q., Wang, S., Duan, H., Sun, C., Guo, Y., Luo, L., Guo, Z., Guan, F., Wang, Z., Xing, A., Liu, Z., Zhang, H., Cui, L., Zhang, L., Jiang, G., Yan, D., Liu, X., Zheng, H., Liang, D., Li, W., Li, Z.C., Zhang, Z.: Neuropathologist-level integrated classification of adult-type diffuse gliomas using deep learning from whole-slide pathological images. Nat Commun. 14, 6359 (2023). doi: 10.1038/s41467-023-41195-9

41. Wang, D., Liu, C., Wang, X., Liu, X., Lan, C., Zhao, P., Cho, W.C., Graeber, M.B., Liu, Y.: Automated Machine-Learning Framework Integrating Histopathological and Radiological Information for Predicting IDH1 Mutation Status in Glioma. Front Bioinform. 1, 718697 (2021). doi: 10.3389/fbinf.2021.718697

42. Zhao, Y., Wang, W., Ji, Y., Guo, Y., Duan, J., Liu, X., Yan, D., Liang, D., Li, W., Zhang, Z., Li, Z.C.: Computational Pathology for Prediction of Isocitrate Dehydrogenase Gene Mutation from Whole Slide Images in Adult Patients with Diffuse Glioma. Am J Pathol. 194, 747–758 (2024). doi: 10.1016/j.ajpath.2024.01.009

43. Farahani, S., Hejazi, M., Tabassum, M., Di Ieva, A., Mahdavifar, N., Liu, S.: Diagnostic Performance of Deep Learning for Predicting Gliomas’ IDH and 1p/19q Status in MRI: A Systematic Review and Meta-Analysis. doi: 10.1007/s00330-025-11898-2

44. Chen, X., Lei, J., Wang, S., Zhang, J., Gou, L.: Diagnostic accuracy of a machine learning-based radiomics approach of MR in predicting IDH mutations in glioma patients: a systematic review and meta-analysis. Front Oncol. 14, 1409760 (2024). doi: 10.3389/fonc.2024.1409760

45. Redlich, J.-P., Feuerhake, F., Weis, J., Schaadt, N.S., Teuber-Hanselmann, S., Buck, C., Luttmann, S., Eberle, A., Nikolin, S., Appenzeller, A., Portmann, A., Homeyer, A.: Applications of artificial intelligence in the analysis of histopathology images of gliomas: a review. npj Imaging 2024 2:1. 2, 1–16 (2024). doi: 10.1038/s44303-024-00020-846. Nobel, S.M.N., Swapno, S.M.M.R., Islam, M.B., Azad, A.K.M., Alyami, S.A., Alamin, M., Liò, P., Moni, M.A.: A Novel Mixed Convolution Transformer Model for the Fast and Accurate Diagnosis of Glioma Subtypes. Advanced Intelligent Systems. 7, 2400566 (2024). doi: 10.1002/aisy.20240056647. Henry, E.U., Emebo, O., Omonhinmin, C.A.: Vision Transformers in Medical Imaging: A Review. https://doi.org/10.48550/arXiv.2211.10043

48. Alleman, K., Knecht, E., Huang, J., Zhang, L., Lam, S., DeCuypere, M.: Multimodal Deep Learning-Based Prognostication in Glioma Patients: A Systematic Review. Cancers (Basel). 15, 545 (2023). doi: 10.3390/cancers15020545

49. D’este, S.H., Nielsen, M.B., Hansen, A.E.: Visualizing Glioma Infiltration by the Combination of Multimodality Imaging and Artificial Intelligence, a Systematic Review of the Literature. Diagnostics 2021, Vol. 11, Page 592. 11, 592 (2021). doi: 10.3390/diagnostics11040592

50. Byeon, Y., Park, Y.W., Lee, S., Park, D., Shin, H.S., Han, K., Chang, J.H., Kim, S.H., Lee, S.K., Ahn, S.S., Hwang, D.: Interpretable multimodal transformer for prediction of molecular subtypes and grades in adult-type diffuse gliomas. npj Digital Medicine 2025 8:1. 8, 1–10 (2025). doi: 10.1038/s41746-025-01530-4
